# Impact of Water Saturation on Microbial Hydrogen Consumption in Porous Media

**DOI:** 10.1021/acs.est.5c08683

**Published:** 2025-12-23

**Authors:** Camille Rolland, Elisabetta Occelli, Myriam Abdelouhabi, Nicolas Jacquemin, Barbora Bártová, Ashley Brown, Olivier Leupin, Rizlan Bernier-Latmani

**Affiliations:** † 27218 Ecole Polytechnique Fédérale de Lausanne (EPFL), Environmental Microbiology Laboratory, Lausanne 1015, Switzerland; ‡ 118537 National Cooperative for the Disposal of Radioactive Waste, Wettingen 5430, Switzerland

**Keywords:** hydrogen, MX80 bentonite, backfill, porous media, deep subsurface, sulfate reducing bacteria (SRB), radioactive waste disposal, deep geological repository (DGR)

## Abstract

Hydrogen (H_2_) production from steel corrosion challenges the long-term stability of deep geological repositories for radioactive waste storage. Subsurface microbial communities are known to consume H_2_, but previous work has primarily focused on fully saturated porous environments. However, gas production will prevent full water saturation of the repository tunnels, where microbial H_2_ consumption is expected. Here, we investigate H_2_ consumption rates at four saturation levels in sand-bentonite, a candidate tunnel backfill material for the Swiss repository concept. The rates ranged from 0.14 μmol·d^–1^·cm^–3^_sand‑bentonite_ (at 70% saturation) to 2.23 μmol·d^–1^·cm^–3^_sand‑bentonite_ (at 90% saturation), exceeding those observed in fully saturated environments. Throughout the 90 days of the experiment, no oxidation was detected below 70% saturation. A sharp rise in H_2_ consumption rate between 70% and 80% saturation reflected the reduced water potential leading to saturation of small pores. This study is the first to examine microbial H_2_ consumption in a natural, partially saturated porous medium, an essential step toward accurately modeling H_2_ sinks as a function of repository saturation. Beyond nuclear waste disposal, our findings may also inform strategies for subsurface H_2_ storage.

## Graphical abstract

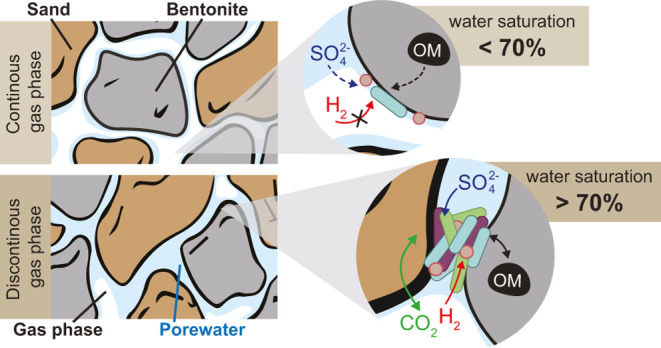

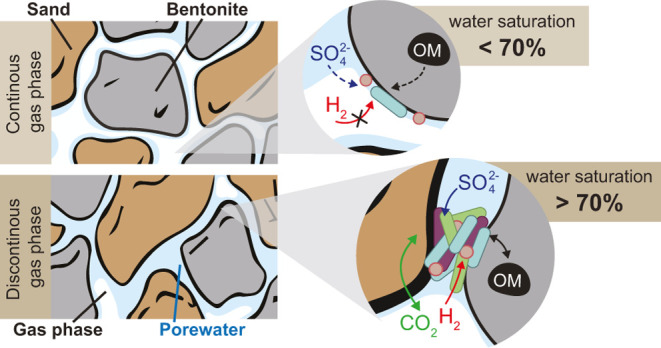

## 1 Introduction

The fate of hydrogen (H_2_) in subsurface environments is a critical concern across strategic domains, including the energy transition,^1^ with underground H_2_ storage^2,3^ or natural resource management,^4–6^ and in the safe disposal of radioactive waste.^7^ In the latter, H_2_ is generated through the anoxic corrosion of steel, and the resulting overpressure presents a long-term risk to the integrity of repositories intended to confine radioactive waste.^8–11^ Depending on the context, the objective may be either to minimize H_2_ consumption, as in H_2_ storage applications, or to maximize it, as in radioactive waste disposal, where reducing gas overpressure is crucial for repository safety.

Radioactive waste disposal often involves the use of clay materials as a host rock and/or a component of the backfill.^12,13^ The backfill plays a key role in regulating gas migration and pressure distribution.^14^ Microbial activity in clay environments is generally low due to restricted space, limited water availability, and scarce organic carbon.^15^ Therefore, microbial processes may be confined to fractures in the excavation damaged zone, or to the backfill, that consists of a material designed to allow sufficient pore space.^16^ Over the past decade, H_2_ has been widely recognized as a key energy source for subsurface microbial communities.^7,17,18^ While clay can sorb or react with limited amounts of H_2_,^19,20^ harnessing microbial H_2_ consumption improves repository safety, and reduces cost.

Recent studies have focused on estimating H_2_ oxidation rates and elucidating the associated microbial metabolisms,^2^ with the goal to predict H_2_ consumption in new locations based on key environmental factors. While temperature has been identified as a major limiting factor,^2,21^ the influence of water availability remains poorly constrained, despite its well-established role in regulating microbial metabolism.^22,23^ To prevent excessive gas buildup, H_2_ consumption must occur prior to full saturation of the backfill. Most studies on microbial H_2_ consumption in radioactive waste disposal settings have focused on fully saturated^24^ or aqueous environments,^25,26^ and do not reflect the expected partially saturated conditions. Therefore, identifying the water saturation threshold for significant microbial H_2_ oxidation is crucial for determining the extent to which the backfill will support hydrogenotrophic communities.

To replicate repository conditions, we adapted traditional microbial cultivation methods and replaced the liquid medium with a porous substrate. We used a mixture (80/20 w) of quartz sand and MX-80 bentonite, the candidate backfill material for the Swiss repository,^14^ partially saturated with Opalinus Clay (the host rock) formation water at 60%, 70%, 80%, and 90% saturation levels. Gas phase monitoring enabled a quantitative assessment of H_2_ consumption. Microbial H_2_ consumption was not observed below 70% saturation, and a threshold in consumption rate emerged between 70% and 80% saturation. Post-experiment biogeochemical characterization of the sand-bentonite matrix suggested that multiple limiting factors, such as water availability, oxygen persistence, and carbon metabolism, may play a role in regulating sulfate-dependent H_2_ oxidation.

Our findings emphasize the importance of suction on microbial activity, an underappreciated factor compared to salinity, pressure and temperature.^2,21^ These results contribute to a more comprehensive understanding of microbial H_2_ consumption in deep subsurface environments and have direct implications for optimizing repository design and gas management strategies in radioactive waste disposal.

## 2 Materials and Methods

### 2.1 Opalinus Clay Formation Water Collection and Characterization

Opalinus Clay formation water was collected under anoxic conditions at the Mont Terri Underground Rock Laboratory (URL) from the BMA-A1 borehole (described in ref 24), and stored in a sterile anoxic and refrigerated jar until use. Duplicate 200 mL samples were filtered with 0.2 μm sterile polycarbonate membrane filters in a flame-sterile environment. The filters were stored frozen in Qiagen LifeGuard solution until DNA extraction. Autoclaved ultrapure water was filtered as a control. DNA was extracted using the phenol-chloroform method (Text S1). For chemical analysis, filtered water was stored in the fridge for quantification of sulfate and organic acids with ion chromatography (Thermo Scientific Integrion HPIC). Ferrous iron was stabilized in 1 M HCl (final concentration 0.5 M) and analyzed using the ferrozine assay.^27^ Sulfide was stabilized in 5% zinc-acetate (final concentration 1%) and analyzed with the Cline assay.^28^ All samples were collected in triplicate (Text S2).

### 2.2 Preparation of Porous Medium and Vials

All equipment was autoclaved (80 min at 120 °C) and preparation was anoxic (N_2_ glovebox). 10 mL crimped gastight vials were filled with 11.5 g of a mix (80/20 w) of quartz sand and MX-80 bentonite (Figure S1), with a controlled grain size distribution (Figure S2), and dry density of 1.5 g·cm^–3^. MX-80 bentonite is a homogeneous material with a well characterized chemical composition.^29^ The material was poured into all vials from the same height without compaction. A top layer of sterile coarse sand was added to maintain a headspace (Figure S1). Water was added by dripping on the surface using sterile needles and syringes to reach the desired saturations (60%, 70%, 80%, and 90%). The mass, density, and water content for each saturation condition are summarized in Tables S1 and S2, the volume of water added for each saturation was calculated as detailed in Text S3. Previous work by Manca et al. has shown that a sand–bentonite mixture with the same grain size and dry density has a bimodal pore distribution (0.2–4 μm and ∼30 μm) at 60% saturation, shifting to a unimodal one (0.2–4 μm) above 70%.^30^ After 1 week of equilibration under N_2_ (20 °C, 0.5 bar overpressure), no wetting front was observed, indicating that the water was homogeneously distributed. This injection method was chosen to improve reproducibility between replicates while mimicking the wetting conditions expected in a repository. The gas phase was replaced with ∼30% H_2_ in N_2_ (0.5 bar overpressure) and the vials incubated at 20 °C for 90 days. The temperature in the Swiss repository is not yet known; therefore, experiments were performed at room temperature, as in previous H_2_ consumption studies applied to the Swiss repository concept.^24,25,31^ The gas phase composition was monitored on a daily to weekly basis, and H_2_ was replenished when undetectable. The vials were stored in a jar with 0.5 bar N_2_ overpressure to minimize leaks. The corresponding total suction for each degree of saturation was measured in a separate test: non-sterile sand-bentonite was partially saturated with artificial formation water (composition in Text S4) and allowed to equilibrate for 1 week; the water content (oven drying at 105 °C for 24 h) and total suction (WP4C Dewpoint PotentiaMeter) were measured (Table S3).

For each degree of saturation (60%, 70%, 80%, and 90%), five vials were prepared: three treatment replicates #1, #2, #3, one abiotic control (autoclaved formation water and sand, gamma-sterilized bentonite), and one H_2_-free vial. Three dry vials were prepared to monitor gas leakage. H_2_-free vials were used exclusively for community analysis (qPCR and 16S rRNA gene sequencing).

### 2.3 Gas Phase Monitoring

Gas composition was analyzed using a Scion 456 gas chromatograph equipped with a two-column system (N_2_ carrier gas), and a Thermal Conductivity Detector for H_2_, CO_2_, O_2_, in series with a Flame Ionization Detector for CH_4_. Triplicate points were taken for the calibration of the instrument to assess the standard deviation (CH_4_ (200 ppm), H_2_ (0.1–2%) and CO_2_ (1%)). For the measurements, 50 μL gas samples were retrieved with a Hamilton gastight syringe using 0.6 mm Sterican needle. The needle was flushed with 10 μL of the sample, and the remaining gas volume was injected in the instrument. Each measurement was repeated only once to limit gas consumption from the vials. Gas sampling frequency was adjusted depending on the gas phase dynamics. H_2_ oxidation rate (% per day) was converted to moles using the ideal gas law (Text S5).

### 2.4 Post-experimental Biogeochemical Characterization

After 3 months, vials were sacrificed in a N_2_ glovebox. Geochemical characterization was performed using (1) aqueous and acid extraction,^32^ and (2) Scanning Electron Microscopy coupled to Electron Dispersive Spectroscopy (SEM-EDS). Aqueous extractions were performed to measure sulfate, and organic acids (formate, pyruvate, lactate, acetate), using ion chromatography (Thermo Scientific Integrion HPIC), and to measure sulfide with the Cline assay.^28^ Acid extraction was performed to quantify bioavailable ferrous and ferric iron using the ferrozine assay.^27^ All extractions were performed in a N_2_ glovebox. For sulfate, 0.75 g of material, was mixed with 250 μL of 1% zinc-acetate, the liquid phase was extracted via centrifugation (10 min at 16,800 rcf), and the supernatant was analyzed. The same extraction protocol was applied for organic acids, replacing zinc-acetate with ultrapure water. For sulfide, 0.2 g of material was extracted with 200 μL of artificial formation water and stabilized via precipitation with zinc-acetate (1%) to prevent oxidation. Iron was extracted from 0.2 g of sand-bentonite with 1 mL of 1 M HCl (24 h equilibration). Extracts were kept in the fridge until analysis. Statistical tests on aqueous extraction results were performed in R (version 4.4.0) using the stats package. Black spots were analyzed for pyrite identification using SEM-EDS. Samples were dried overnight in a N_2_ glovebox, transported in Mylar bags, and analyzed using a Zeiss Gemini-SEM 300 microscope under high vacuum without coating. Framboids were visually identified, and EDS was used to determine the iron:sulfur ratio.

### 2.5 DNA Extraction from Sand-Bentonite

DNA was extracted from triplicate samples from each vial. A novel in-house DNA extraction protocol was developed (patent pending), incorporating deflocculation with sodium-hexametaphosphate alongside the chemical and mechanical lysis steps of the DNeasy Power Soil Pro Kit (Qiagen). This new protocol yields 100 times more DNA than the unmodified commercial kit, making it a competitive alternative to the labor-intensive phenol-chloroform method^33^ (Table S4 for detailed yield comparison). Briefly, the manufacturer’s protocol was modified as follows: 200 μL of 10% sodium-hexametaphosphate (buffered at pH 8 with NaHCO_3_) was added simultaneously with CD1 solution to 0.2 g sand-bentonite sample. Samples were vortexed, incubated at 65 °C for 10 min, and bead-beaten using the Precellys 24 (Bertin) at 5,000 rpm for 2 × 20 s. Following the addition of CD2 (purification step), the entire supernatant volume was recovered and loaded onto the spin column. Finally, the elution buffer (C6, 60 μL) was amended to the spin column and allowed to incubate for 10 min at room temperature prior to collection by centrifugation. DNA concentration was measured using the Qubit 1x dsDNA HS Assay Kit. Each extraction batch included the hexametaphosphate improved kit-only extraction control. No bacterial or archaeal contamination was detected (Figure S3).

### 2.6 Quantitative PCR Analysis

Quantification of the number of bacteria and archaea 16S rRNA genes was performed in triplicate using respectively the primers 338f (5′-ACTCCTACGGGAGGCAGCAG-3′)^34^/520r (5′-ATTACCGCGGCTGCTGG-3′)^34^ and 931f (5′-AGGAATTGGCGGGGGAGCA-3′)^35^/M1100r (5′-BGGGTCTCGCTCGTTRCC-3′).^36^ 10 μL reactions were prepared on a MYRA robotic system and run on a MIC qPCR Cycler (BioMolecular Systems, Australia): 2.5 μL template DNA, 2.1 μL water, 0.2 μL of each primer (10 μM stock) and 5 μL of 2xSensiFAST SYBR No-ROX Kit (Bioline, England). Samples were cycled (x40) at 95 °C for 5 s, followed by extension at 62 °C for 15 s and acquisition at 72 °C for 15 s. The final melting step was performed from 72 to 95 °C, at a rate of 0.1 °C.s^–1^. Analysis of the results was performed using the integrated analytical software (micPCR, BioMolecular Systems, Australia). Efficiency (0.82–1.04 and *r* ^2^ values (>0.995)) was determined from eight points of serial dilution (10^8^–10^1^ copies) of the target gene. The effect of saturation and H_2_ on archaea and bacteria abundance was assessed using Kruskal–Wallis and Student *t* tests, p-values were corrected with the Benjamini-Hochberg method.

### 2.7 16S rRNA Full-Length Gene Sequencing

Amplicons were prepared following the PacBio procedure: 14 μL of 1x KAPA HiFi HotStart ReadyMix, 6 μL of forward and reverse primers, and 5 μL of sample. Samples with a DNA concentration above 0.5 ng·μL^–1^ were normalized to 0.5 ng·μL^–1^ using the C6 elution buffer (Qiagen). Ninety-six combinations of primers were provided by the Lausanne Genomic Technologies Facility (Switzerland). Samples were run on a thermocycler with an initial denaturation at 95 °C for 3 min, 25 cycles of 30 s at 95 °C for denaturation, 30 s at 57 °C for annealing, and 60 s at 72 °C for extension. The 16S rRNA gene degenerate forward primers sequence was GCATC/barcode/AGRGTTYGATYMTGGCTCAG, and the 16S rRNA gene degenerate reverse primers sequence was GCATC/barcode/RGYTACCTTGTTACGACTT. Equimolar pooling and quality checks were performed by the Bern Next Generation Sequencing platform for sequencing with the Revio 84151 on one SMRT cell. Sequence quality check was performed with FastQC (v0.11.9), dereplication, error modeling, and ASV inference with DADA2 (v1.30.0),^37^ taxonomic classification using the RDP Classifier with the SILVA database (release 138.2),^38^ and functional prediction of metabolic pathways using PICRUSt2 (v2.5.0).^39^ Relative abundances were adjusted based on KEGG orthology. A phyloseq object^40^ was created to remove singletons (filter_taxa function, validation with microViz package^41^). Data were transformed using the Hellinger function (microbiome package), and the Bray–Curtis dissimilarity. The samples were clustered with the Ward.D2 method (NbClust package,^42^ cophenetic correlation coefficient of 86.4), using silhouette index and permutation testing (adonis2, vegan package^43^). Cluster prediction was performed using a Conditional Inference Tree (ctree function, party package^44^), with saturation level and presence of H_2_ as predictors. Distance-based redundancy analysis (dbRDA) was used to model the data (model verified with anova.cca function, vegan package^43^). PICRUSt2 results were processed using ggpicrsut2 package.^45^

## 3 Results and Discussion

### 3.1 Sulfate Reduction Driven H_2_ Oxidation

The concentrations of H_2_, CH_4_, CO_2_ and O_2_ in the treatment vials were monitored to quantify the H_2_ consumption rate as a function of water availability, and to probe for metabolic activity. O_2_ was never detected. H_2_ concentration was stable in the dry controls, demonstrating negligible leakage (Figure S4). At 25% H_2_ in the gas phase, dissolved H_2_ accounts for about 5% of the total H_2_ at 60% water saturation and 10% at 90%. Dissolution effect on gas phase concentration appears in the abiotic controls as a 5–10% H_2_ drop during the first ∼10 days (Figures S5–S8). The later stable H_2_ partial pressure in the abiotic controls indicated that the H_2_ consumption observed in the treatment vials was biologically driven (Figures S5–S8).

Significant H_2_ consumption started in the three vials at 90% saturation between 20 and 30 days into the experiment (Figure S8). In vials #2 and #3 at 80% saturation, consumption started within the same time frame (Figure S7). In these vials, full H_2_ consumption is observed after 40 days (Figure 1A). The initial 35 days of the experiment correspond to a microbiome establishment phase; therefore, only data collected after this period were included in the subsequent analysis. Linear rates were calculated for each H_2_ injection interval (Figure 1B, Table S5). H_2_ consumption rates increased with water availability, showing a sharp and significant rise when water saturation is at or above 80%. The average rate was 0.63 μmol·d^–1^·cm^–3^_sand‑bentonite_ in 90% vials, and 0.40 μmol·d^–1^·cm^–3^_sand‑bentonite_ in 80% vials #2 and #3. In the 80% vial #1 and 70% vial #3, H_2_ consumption was observed (0% remaining after 70 days, Figure 1A), although the consumption rate was slower (0.08 μmol·d^–1^·cm^–3^_sand‑bentonite_). In contrast, in the other 70% vials (#1 and #2), and at 60% saturation, it was difficult to distinguish gas phase changes from leakage (i.e., dry and abiotic vials, Figure 1B). The variable behavior among triplicate vials at 70% and 80% saturation suggests a threshold for microbial H_2_ consumption within this saturation range.

**Figure 1:**
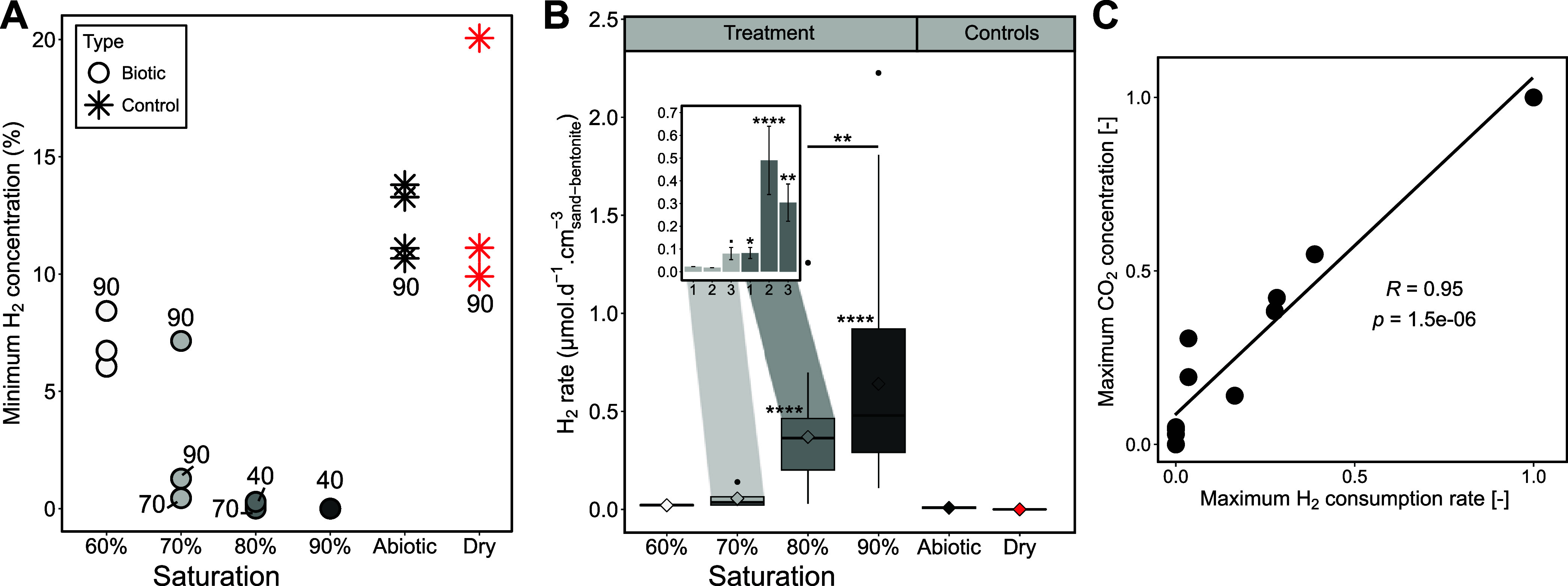
H_2_ consumption in treatment and control vials. (A) Minimum H_2_ concentration recorded in each vial, along with the earliest day at which it was observed, indicated by the number associated with each marker. (B) Box plot illustrating H_2_ consumption rate in vials at different saturation levels, excluding the first 35 days. The central line of each box represents the median, and the box edges correspond to the first and third quartiles. Whiskers extend to the minimum and maximum values within 1.5 times the interquartile range, while individual points beyond this range are plotted as outliers. The mean is indicated by a diamond inside the box. Stars at boxes edges indicate statistically significant differences compared to the controls, and stars between boxes, statistical difference between saturation levels. Consumption rates at 80% and 90% saturation were statistically different from the controls (Welch test, ****: *p* < 0.0001), and 90% was significantly different from 80% (**: *p* < 0.01). The inset plot details the rate for each vial at 70% and 80% saturation, bar height represents the mean, error bars indicate one standard deviation, stars indicate whether the rate is significantly different from the controls (•: *p* < 0.1, *: *p* < 0.05). (C) Correlation (linear regression) between H_2_ consumption rate (μmol·d^–1^·cm^–3^_sand‑bentonite_) and CO_2_ concentration (%), with each point representing a treatment vial. Data were normalized (min-max) prior to the regression and are presented unitless.

The experimentally determined H_2_ oxidation rates in this study ranged from 0.14 (70% saturation) to 2.23 (90% saturation) μmol·d^–1^·cm^–3^_sand‑bentonite_. These rates are consistent with the H_2_ oxidation rates previously derived from sulfate reduction in sand-bentonite saturated with Opalinus Clay formation water: 0.9–3.2 μmol·d^–1^·cm^–3^_sand‑bentonite_.^24^ While water promotes growth (shown by a higher abundance at the end of the experiment, later described), higher mass transfer between gas and liquid in partially saturated porous medium can increase the observed consumption rates.^46^ To facilitate comparison with other studies, rates were normalized to the volume of water present in the vials (Text S5, Table S2). Average rates ranged from 0.25 μmol·d^–1^·cm^–3^_water_ (70% #3 and 80% #1) to 1.26–1.82 μmol·d^–1^·cm^–3^_water_ (90% saturation), while maximum rates reached 3.3–5.5 μmol·d^–1^·cm^–3^_water_. These rates are higher than previously reported for sulfate-dependent H_2_ consumption: Bagnoud et al. reported H_2_ oxidation rates *in situ* in Opalinus Clay formation water, ranging from 0.72 to 1.37 μmol·d^–1^·cm^–3^ which was twice as high as what could be stoichiometrically accounted for by sulfate reduction,^25^ and Dohrmann & Kruger reported 0.123–0.325 μmol·d^–1^·cm^–3^ for sulfate-derived H_2_ oxidation.^47^

Potential microbial H_2_ sinks include sulfate reduction, iron reduction, methanogenesis, and homoacetogenesis.^2,7^ CH_4_ was measured in vials around a few hundred ppm (Figure S9), which is at the limit of quantification for the instrument used. The presence of CH_4_ in abiotic vials in the same range as in treatment vials suggests (conjugation mistake) its origin from the clay formation water. CO_2_ was not detected in any of the abiotic vials, while in treatment its concentration clearly increased over time under wetter conditions (Figures S10A and S10B) and showed a positive correlation with H_2_ consumption (linear regression, *R* = 0.95, *p* < 0.0001, Figure 1C). The variation in CO_2_ concentration due to H_2_ consumption or changes in total pressure cannot account for the continuous accumulation of CO_2_, thus the increase in CO_2_ concentration is interpreted as being due to biological activity. Up to 2.5–5% CO_2_ was detected in 80% saturation vials #2 and #3 and up to 5–14% in 90% saturation vials (Wilcoxon test, *p* < 0.005 compared to abiotic). Slight production was also observed in vials where H_2_ consumption was slower but noticeable (i.e., 70% #3 (*p* = 0.014) and 80% #1 (*p* = 0.008)).

After 90 days, the final concentrations of sulfate, sulfide, acetate, as well as ferrous and ferric iron were measured as indicators of microbial activity (Figure 2A-C, Figure S11). Lower sulfate concentrations were observed in all treatment vials compared to the abiotic controls (*p* < 0.05, Figure 2A). This suggests that limited sulfate reduction may still occur at lower saturations. The 80% and 90% vials exhibited lower sulfate concentrations than the 60% and 70% vials, except for vial 70% #3, in which H_2_ consumption was also observed. While the final sulfate concentration depends on water availability (Kruskal–Wallis test, *p* = 0.048, Figure 2A) a stronger negative correlation was observed with H_2_ consumption (*R* = −0.6, *p* = 0.038, Figure S12), confirming that H_2_ metabolism is associated with sulfate consumption. Sulfate reduction results in sulfide production, however, sulfide concentration was highest in the 60% vials. Both water content (*p* = 0.023, Figure 2B) and H_2_ consumption (*R* = −0.64, *p* = 0.026, Figure S12) were negatively correlated with sulfide. Increased water availability likely promotes the formation of readily accessible sulfide sinks, whereas reduced diffusion under lower water content may restrict their availability. Extracted ferric or ferrous iron concentrations were not correlated with water content but an overall increase of extractable ferrous iron (the predominant form of iron) was observed in all treatment vials relative to initial and abiotic conditions (grammar error) (Kruskal–Wallis test, *p* < 0.01, Figure S11). Starting from week 6 of the experiment, the formation of black spots was observed in vials at 90% saturation (Figure S13) and later also in the 80% vial #1. When characterized by SEM-EDS, these black spots revealed the presence of framboidal pyrite in 90% vials #2 and #3 (Figure S14 and Figure 3), indicating that iron sulfide precipitation acts as a sink for sulfide. The iron to sulfur ratio was around 0.5 in six out of the eight framboids observed, which corresponds to pyrite (FeS_2_). Two iron-sulfide precipitates with peculiar morphologies were observed. One had a much lower iron to sulfur ratio (0.33, Figure S14A), while the other had larger and more angular crystals (Figure S14F) and an iron to sulfur ratio of 0.75, potentially corresponding to greigite (Fe_3_S_4_), known to be an intermediate in framboidal pyrite formation.^48^ However, because the signal penetration depth is approximately 1 μm, contributions from underlying materials may have influenced the measured ratios. Acetate was below the initial acetate concentration (∼1 μmol·g^–1^), or near the limit of detection in the vials with 60% and 70% saturation (except vial 70% #3) and was detected at significantly higher concentrations in vial 70% #3, and all vials with 80% and 90% saturation (*p* < 0.0001, Figure 2). Acetate production was positively correlated with H_2_ consumption (*R* = 0.66, *p* = 0.02, Figure S12). Other organic acids (formate, pyruvate, lactate) were not detected. Detailed Student *t* test results for sulfate, sulfide, and acetate are presented in Table S6.

**Figure 2:**
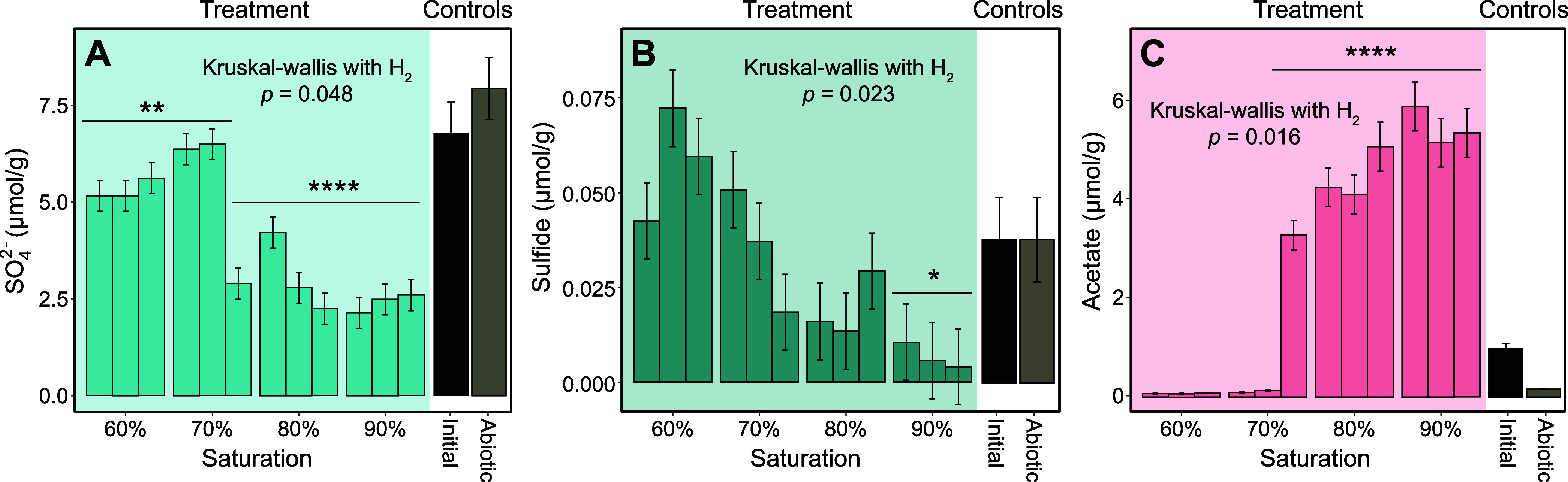
Sulfate, sulfide, and acetate content post-incubation. (A) Sulfate, (B) sulfide, and (C) acetate concentrations in extractant from treatment with H_2_ (left to right: vial 1, 2, and 3 for each degree of saturation) and control vials (average for all saturations). Error bars represent technical replicates. Kruskal–Wallis test results on the effect of saturation in treatment vials are shown: in the presence of H_2_, saturation significantly affects all three compounds. Statistically significant differences in comparison with the abiotic setup are indicated (*: *p* < 0.05, **: *p* < 0.01, ****: *p* < 0.0001). Detailed *t* test results are provided in Table S6.

**Figure 3:**
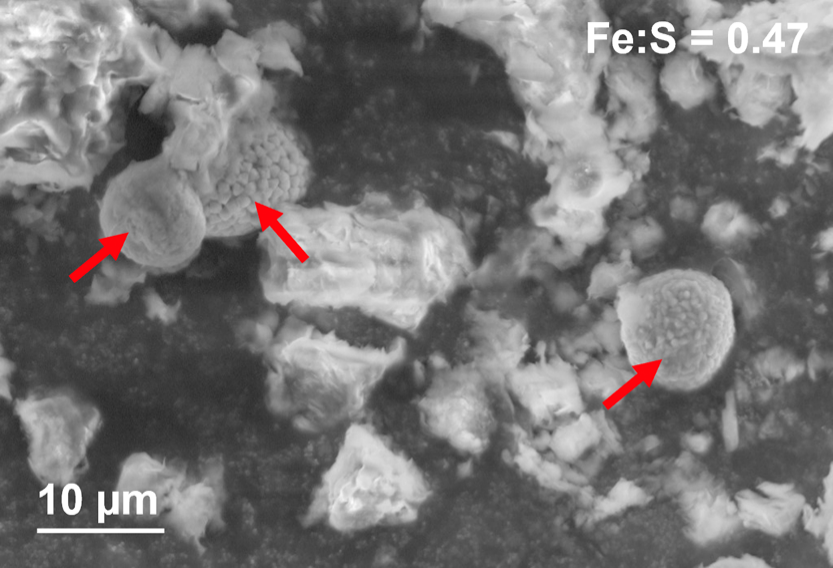
Framboidal pyrites. SEM image of three iron-sulfide precipitates (arrows) observed in vial 90% #3. EDS analysis of the three framboids showed an average Fe:S atomic ratio of 0.47.

In the presence of H_2_, bacterial abundance (Figure S15A) was significantly correlated with water availability (Kruskal–Wallis test, *p* < 0.001), while in the absence of H_2_, no significant correlation was observed (*p* = 0.09). With H_2_, bacterial abundance was higher at 70% saturation as compared to 60% (*p* < 0.01), and higher above 80% as compared to lower saturations (*p* < 0.001). However, no difference in biomass was detected between the 80% and 90% saturation conditions (*p* = 0.36). Bacterial abundance showed a weak correlation with H_2_ consumption (*R* = 0.53, *p* = 0.075, Figure 4C). When comparing bacterial abundance with and without H_2_, H_2_ had a significant impact at 90% saturation (*p* < 0.001), while at 80% saturation, only vial #1 showed significantly higher abundance with H_2_ (*p* < 0.001), and at 60% and 70% saturation, there was no significant difference. In both treatment and abiotic vials archaeal abundance was minimal (Figure S15B), four orders of magnitude lower than that of bacteria. Overall, neither H_2_ nor water availability promoted the growth of archaea. Only 90% saturated H_2_-free vial exhibited significantly different behavior (*p* = 0.03) with a clear increase in archaeal abundance. Detailed results for the Wilcoxon tests are presented in Table S7.

**Figure 4:**
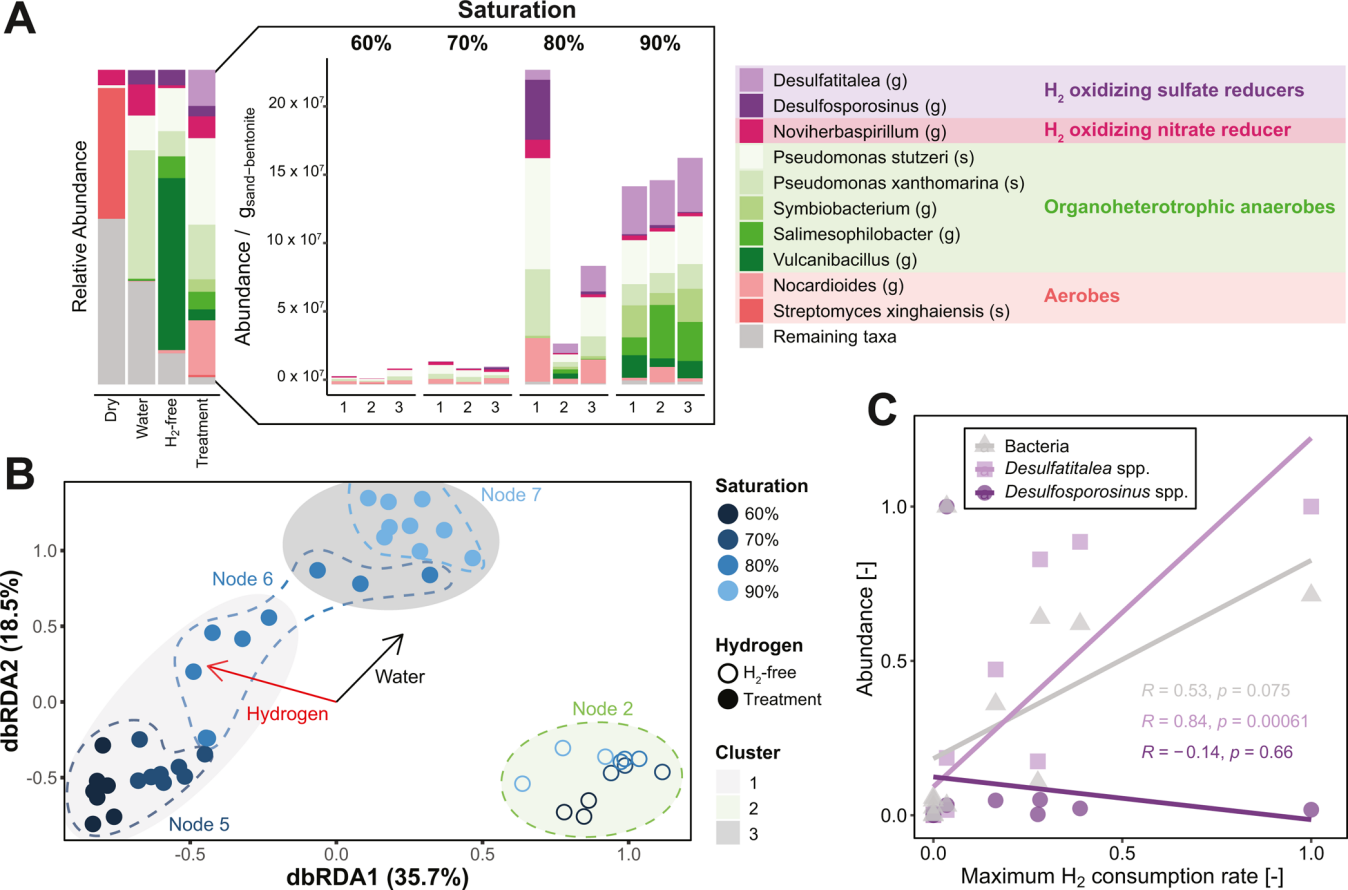
Microbial community in the treatment and control vials. (A) (Left) Relative abundance in the initial material (dry sand-bentonite and Opalinus Clay formation water), in the H_2_-free control, and in the treatment vials. (Right) Abundance in each treatment vial, calculated from relative abundance and bacterial biomass: $\mathrm{Abundance}=\frac{n_{\mathrm{reads}}}{n_{\mathrm{reads},\mathrm{total}}}\times \frac{\mathrm{bacterial}16S\;\mathrm{rRNA}\;\mathrm{gene}\;\mathrm{copies}}{\mathrm{sand‐bentonite}\;\mathrm{sample}\;\mathrm{mass}}$ . The average of triplicate extractions per vial is represented. (B) Distance-based redundancy analysis (dbRDA) conducted with Bray–Curtis dissimilarity on Hellinger transformed relative abundance (triplicate samples per vial). Both axes are significant (*p* < 0.001) with the percentage of variance indicated. The samples were hierarchically grouped into three clusters, and significant threshold levels for H_2_ and water availability in shaping the tree structure were determined using a Conditional Inference Tree. (C) Correlation (linear regression) of H_2_ consumption rate (μmol·d^–1^·cm^–3^_sand‑bentonite_) with bacterial abundance (16S rRNA copies g^–1^_sand‑bentonite_) and sulfate reducers abundance (*Desulfatitalea* spp. and *Desulfosporosinus* spp.). Each point represents a treatment vial. Data were normalized (min-max) prior to the regression and are presented unitless.

Microbiome alpha diversity (Figure S16) was calculated using the Shannon index, which considers both species richness and evenness. Higher water availability led to increased diversity in both treatment vials (*p* < 0.001) and H_2_-free controls (*p* = 0.02). The range of the Shannon index was larger in treatment vials (1.6–2.9) compared to H_2_-free controls (2–2.5). Detailed results for the Wilcoxon tests are presented in Table S8. Distance-based redundancy analysis (*p* < 0.001, adjusted *R* ^2^ = 0.52) indicated that both H_2_ and the degree of saturation significantly shaped the microbiome (*p* < 0.001). The samples were grouped in three clusters (Figure 4B and Figure S17A). The affiliation of samples to these clusters was predicted based on H_2_ and water availability using a Conditional Inference Tree (Figure S17B). The tree identified three significant levels of separation, resulting in four nodes: three pure nodes (each holding a single cluster) and one mixed node (containing samples from clusters 1 and 3). The first level of separation was associated with the presence or absence of H_2_ (*p* < 0.001, node 2/cluster 2 in Figure 4B), the second level with a saturation degree of 90% (*p* < 0.001, node 7 in cluster 3), and the third level with a saturation degree below 80% (*p* = 0.035, node 5 in cluster 1). The samples from the 80% vials (node 6 in Figure 4B) split in cluster 1 for vials #1 and #3 (along with 60% and 70% vials), and in cluster 3 for vial #2 (with 90% vials). Therefore, while the communities at 60–70% saturation, and 90% saturation, were self-consistent, samples from 80% saturation vials formed a transition group.

In the presence of H_2_, increased water availability leads to a shift in the microbial community (Figure 4A), with the growth of hydrogenotrophic sulfate reducers (a third of the community at 90% saturation), such as *Desulfatitalea* spp. (only present with H_2_), and *Desulfosporosinus* spp. (present in treatment vials and H_2_-free controls). The growth of *Desulfatitalea* spp. shows a strong positive correlation with H_2_ consumption (R = 0.84, *p* < 0.001, Figure 4C), while no correlation is observed for *Desulfosporosinus* spp. *Noviherbaspirillum*, a nitrate-reducing genus capable of oxidizing H_2_, but also able to grow under aerobic conditions,^49^ is almost absent without H_2_, and grew the most in 80% vial #1, yet its relative abundance decreases with water availability (Figure S18). Nitrogen was predominantly present as dinitrogen gas, with no detectable nitrate, nor nitrite. Thus, oxidation of H_2_ via nitrate reduction did not contribute, or contributed only minimally, to H_2_ removal. At higher water saturation, a decrease of the relative abundance of aerobes is observed, and their abundance shows a weak negative correlation with the H_2_ consumption rate (R = −0.14, *p* = 0.66, Figure S19). At 90% saturation with H_2_, anaerobic organoheterotrophs constitute two-thirds of the microbial community. *Pseudomonas stutzeri* and *Pseudomonas xanthomarina*, which exhibit a sequence similarity of over 98%^50^ and harbor metabolically versatile strains,^51^ are dominant in the Opalinus Clay formation water, and in sand-bentonite across the entire saturation range (Figure 4A). In contrast, *Salimesophilobacter, Vulcanibacillus,* and *Symbiobacterium* genera presence falls below 10% in vials #2 and #3 at 80% saturation and remains undetectable in vial 80% #1 and at lower saturation levels. On the contrary to *Pseudomonas* spp, their growth is significantly proportional to H_2_ consumption (R = 0.85, *p* < 0.0001, vs R = 0.27, *p* = 0.4, Figure S19). *Salimesophilobacter* spp. use sulfur, thiosulfate and ferric iron as electron acceptors but not sulfate,^52^ *Vulcanibacillus* spp. need nitrate,^53^ and *Symbiobacterium* spp. reduce nitrate and DMSO.^54,55^ No identifiable homoacetogens nor methanogens were detected.

The correlation between the rate of H_2_ oxidation with both the remaining sulfate concentration (Figure S12), and the growth of sulfate reducers (Figure 4C) support the hydrogenotrophic activity of sulfate-reducing bacteria. Furthermore, the formation of framboidal pyrite has been suggested to depend on the presence of organic matter,^56^ and is considered to be a potential marker of biogenic iron-sulfide formation.^57^ The observed decrease in sulfide concentration, despite being the end-product of sulfate reduction, is attributed to its precipitation with ferrous iron. In the presence of H_2_, the ferrous iron concentration was higher across all saturations compared to controls, pointing to ferric iron reduction. Whether it is enzymatic or due to an abiotic reaction with sulfide^58^ is unclear. Despite the availability of CO_2_ in some vials, methanogenesis was not observed, which aligns with the absence of methanogenic archaea in the sequencing results and with previous studies suggesting that methanogens are outcompeted by sulfate reducers.^59–63^ As methanogenesis has a slightly higher Gibbs energy than homoacetogenesis (respectively −43.9 kJ mol^–1^ H_2_, and −36.1 kJ mol^–1^ H_2_, under standard conditions^64^), the production of acetate is unlikely to result from homoacetogenesis. Instead, both acetate and CO_2_ likely originate from the degradation of organic carbon potentially originating from bentonite.^65^

The key role of sulfate reducers in H_2_ oxidation has been previously observed in similar subsurface environments.^24,25,60^ However, in this study, a distinct pattern emerges between the two sulfate-reducing genera present. There is a strong correlation between *Desulfatitalea spp.* abundance and H_2_ oxidation rate, while porous media dominated by *Desulfosporosinus spp.* exhibit lower H_2_ consumption (Figure 4C). Some species within the *Desulfosporosinus* or *Desulfatitalea* genera can use both H_2_ and organic carbon, including acetate, as electron donors, or grow on H_2_ and CO_2_.^66–68^ In vials where CO_2_ was produced, H_2_ consumption was more rapid (Figure 1C), with *Desulfatitalea* spp. outcompeting *Desulfosporosinus* spp. (Figure 4A,C). This suggests that *Desulfatitalea* spp. may utilize H_2_ and CO_2_ more efficiently, providing a competitive advantage when CO_2_ accumulates. Thus, CO_2_ availability could be a rate limiting factor for H_2_ consumption. Conversely, the activity of heterotrophs capable of degrading complex organic carbon can stimulate the mixotrophic metabolism of hydrogenotrophs, as suggested by the positive correlation of H_2_ oxidation rate with the growth of heterotrophs (Figure S19). Further, PICRUSt analysis (Figure S20) indicates that pyruvate fermentation to acetate occurs across all water saturations in the absence of H_2_, while in the presence of H_2_, this fermentation pathway requires H_2_ saturation above 80%. Indirectly, this suggests that fermenters were limited by the sustained high concentration of H_2_ at 60–70% saturation. Elevated H_2_ concentrations in the gas phase may impose constraints on the microbial community: for instance, a reduced alpha diversity was observed for a same saturation in treatment vials as compared to H_2_-free vials (except at 90% saturation) (Figure S16). H_2_ consumption may favor fermentation, and might create a positive feedback loop, where fermentation promotes further H_2_ consumption by producing low molecular weight carbon compounds. The potential for carbon cycling in a repository with persistently high H_2_ concentrations in the gas phase warrants further investigation.

### 3.2 Water Saturation Threshold for Microbial H_2_ Consumption

Determining the water saturation threshold for microbial H_2_ consumption is essential for estimating the volume of backfill material capable of sustaining microbial activity at different stages of repository saturation. While minimal growth and metabolic activity (e.g., sulfate consumption and acetate production) occur at 60–70% saturation, a distinct threshold for microbial growth and H_2_ metabolism emerges between 70% and 80% saturation.

Water availability is typically assessed using water activity (a_w_ = *p*/*p* ^0^) where *p* is the vapor pressure in the system and *p* ^0^ the vapor pressure at equilibrium with pure water. A water activity of 0.96 is generally considered the lower limit for the survival of most Gram-negative bacteria.^22^ While this threshold has been validated in liquid systems where water availability is controlled by salt concentration, its application has been overgeneralized to porous materials.^69,70^ In soil-like systems, including clay-rich porous media, water availability is described in terms of water potential also referred to as total suction (ψ), which combines solute potential (ψ_s_) and matric potential (ψ_m_): $\psi =\psi_{s}+\psi_{m}=-\frac{RT\rho }{\omega }\ln\left(a_{w}\right)$ ^71^ where *R* is the universal gas constant, *T* the temperature, ρ the density of water, and ω the molecular mass of water. ψ_s_ represents the contribution from salts, while ψ_m_ accounts for capillary forces. While previous research on microbial activity in bentonite reported a water activity threshold of 0.96 (corresponding at ambient temperature to a total suction of 5.1 MPa),^69,70^ a more detailed analysis indicates that this value might be insufficiently restrictive. In contrast, it has been recognized for over 50 years that environments dominated by matric potential may exhibit a higher water potential limit for microbial activity. Factors such as water movement and pore connectivity play crucial roles in determining microbial viability. As a result bacterial activity in soils is significantly constrained at water potentials higher than those required to inhibit growth in liquid media.^23^ As previous studies have shown that bentonite microorganisms can become active when provided with water and nutrients,^72^ the primary concern in the context of assessing microbial H_2_ oxidation is not microbial survival, but rather the rate of metabolism.

A soil mechanics characterization of sand-bentonite (80/20 w) by Manca et al.^14^ reports that for a material compacted at 1.5 g.cm^–3^, a water activity of 0.96 corresponds to a saturation of 20%, which is well below the threshold for microbial H_2_ consumption observed here (Figure S21). Our measurements of total suction across the saturation range investigated revealed that saturation levels between 74% and 81% correspond to total suction values of 2–1.6 MPa, which equates to a water activity range of 0.986–0.988 (Table S3). These findings suggest that in partially saturated systems, the commonly referenced water activity threshold of 0.96 significantly underestimates the actual limit for significant microbial growth and metabolism.

To explain this threshold, we examined data from experimental dynamic compaction tests on sand-bentonite from Manca^14^ (Figure S22). The 70–80% saturation range corresponds to the shift from a two-phase system, with a continuous gas phase, to the occurrence of local sites of water-filled porosity. At lower saturation levels, suction is dominated by capillary forces, and as saturation passes the two-phase threshold, water pockets form, enhancing water availability and supporting microbial growth. At higher saturations, the increase in the size of water pockets enhances the volume of soil accessible to microbial colonization, which explains the difference observed here between 80% and 90% saturation. The variability observed for microbial growth at 80% saturation may be attributed to sampling discrepancy or heterogeneity in the distribution of water pockets.

While the increase in water availability, corresponding to a sharp decrease in suction forces, is likely responsible for the increase in microbial growth, H_2_ consumption was not proportional to this growth (e.g., in 80% vial #1 and 70% vial #3). This suggests that water availability is not the only factor driving the observed trend. In this experiment, strictly anaerobic sulfate reducers are hypothesized to be the main and fastest H_2_ consumers. Therefore, an explanation for the lack of H_2_ consumption at 60–70% saturation could be the persistence of oxygen. With limited growth, microaerophilic conditions likely dominate, preventing anaerobic H_2_ consumption. However, as water pockets filled, anoxic microsites^73^ would quickly establish, enabling efficient H_2_ consumption by sulfate reducers. Over time, depletion of oxygen could allow H_2_ consumers to thrive even at lower saturations. Furthermore, water saturation and gas transport are inversely related.^14^ Thus, above 80% saturation, when the gas phase is not continuous, H_2_ availability may be limited by its dissolution rate.

## 4 Environmental Implications

This study provides valuable insights into the potential for microbial H_2_ oxidation within engineered backfill materials. Our results indicate that complete saturation is not a prerequisite for microbial activity. These findings reinforce the crucial role of microbial processes in subsurface environments as it highlights their adaptability to varying hydration conditions. Predicting the volume of filled porosity will be most relevant to estimate the rate of microbial H_2_ consumption in the subsurface.

Higher consumption rates in comparison with previous studies underscore the importance of studying microbial activity in relevant geological environments rather than relying solely on experiments in liquid environments. While absolute comparisons with previous work remain difficult due to differences in water and H_2_ availability, the fact that our results fall within the same order of magnitude strengthens the reliability of these rates for repository modeling.^9,16^

## Supplementary Material



## Data Availability

The data sets generated and analyzed for this study can be found in the Zenodo repository at: 10.5281/zenodo.15752663 All scripts and Dockerfiles used for the analysis are available at: https://github.com/nlmjacquemin/emlexp108109110

## Notes

Detailed information on methods, including experimental setup description and DNA extraction; bacterial and archaeal biomass quantification plots; detailed gas-phase evolution for H_2_, CO_2_, and CH_4_; complementary post-experimental chemistry composition plots, including correlation with H_2_ consumption rate; additional SEM-EDS microstructural and elemental composition analysis; alpha-diversity plot; hierarchical clustering results; taxa relative abundance in treatment vials, correlation of H_2_ consumption rate with the abundance of aerobes and non-H_2_ oxidizing heterotrophs; PICRUSt functional inference results; water retention behavior of sand-bentonite; H_2_-consumption rate table; detailed statistical analysis outputs (PDF)
