# Supplementary material for: Impact of Water Saturation on Microbial Hydrogen Consumption in Porous Media
Source: Environ Sci Technol. 2025 Dec 23;60(1):415–24. doi: 10.1021/acs.est.5c08683 (PMC12810378; doi:10.1021/acs.est.5c08683)
Supplement: Supplementary file 1 [file es5c08683_si_001.pdf]

Supplementary Information

Impact of Water Saturation on Microbial Hydrogen Consumption in Porous Media

Camille Rolland[1], Elisabetta Occelli[1], Myriam Abdelouhabi[1], Nicolas Jacquemin[1], Barbora Bártová[1], Ashley Brown[2], Olivier Leupin[2], and Rizlan Bernier-Latmani[1*]

[1]Environmental Microbiology Laboratory, École Polytechnique Fédérale de Lausanne, Lausanne, Switzerland

[2]National Cooperative for the Disposal of Radioactive Waste, Wettingen, Switzerland

*Corresponding author: rizlan.bernier-latmani@epfl.ch

Number of pages: 23

Number of figures: 21

Number of tables: 5

1. Supplementary text 1
2. Supplementary figures 3
3. Supplementary tables 18

1. Supplementary text

Text S1. DNA extraction from filters.

Cell Lysis:

1. Filters were transferred to sterile bead-beating tubes (Qiagen) and processed using a Precellys 24 homogenizer at 5,000 rpm for 2 x 20 s.
2. Added 1.3 mL TE buffer (pH 8) and 65 µL lysozyme (150 µg/L), vortexed for 10 min, and incubated at 37°C for 2 h.
3. Added 26 µL of sterile 5% SDS, followed by three cycles of cold shock (dry ice/ethanol) and heat (90°C water bath) for 5 min each.
4. Added 14 µL proteinase K (200 µg/mL) and incubated at 55°C for 2 h.

Phenol-Chloroform Purification and DNA Precipitation:

1. Samples were extracted with phenol/chloroform/isoamyl alcohol, centrifuged at 14,000 g for 1 min, and the aqueous phase was collected (repeated x3).
2. Further purified with chloroform/isoamyl alcohol (1:1), followed by ethanol precipitation using 100% ethanol, 0.1 volume of 3M sodium acetate (pH 5.2), and 1 µL linear polyacrylamide (25 mg/mL).
3. DNA pellets were washed with 70% ethanol, air-dried, and resuspended in 40 µL TE buffer (pH 8), incubated at 60°C for 15 min.

Text S2. Opalinus Clay formation water composition.

The water has a pH of 7.2 ± 0.1 and contains 35 ± 2 µmol/L of ferrous iron, with no detectable ferric iron and sulfide. The concentrations of dissolved inorganic and organic carbon are respectively 28.7 ± 1.4 mg/L and 4.2 ± 3.1 mg/L. Major ions include sulfate (16 ± 4 mM), calcium (13 ± 1 mM), magnesium (14 ± 1 mM), sodium (211 ± 5 mM), chloride (245 ± 5 mM), bromide (383 ± 12 µM), ammonium (1.0 ± 0.2 mM), and potassium (1.35 ± 0.03 mM).

Text S3. Degree of saturation.

The degree of saturation DS_r was calculated as follow:

$$DS_r(\%) = \frac{V_{water}}{V_{voids}} \times 100 \quad \text{with} \quad V_{voids}(cm^3) = V_B(cm^3) - V_S(cm^3) = V_B - \frac{M_S\,(g)}{G_S[-] * \rho_{water\,(\frac{g}{cm^3})}}$$

With, $V_B$the bulk volume, V_S the solid volume, M_S the dry mass, and G_S the specific gravity of the material (2.67 for 80/20 sand/bentonite [1]). The density of the material is obtained by multiplying the specific gravity by the density of water (0.997 g/cm^3). Numerical values for each saturation conditions are summarized in Table S1.

Text S4. Artificial formation water composition.

The artificial formation water had a pH of 7.2, and a composition of 250 mM sodium, 300 mM chloride, 27 mM sulfate, 2.3 mM potassium, 30 mM calcium, and 11 mM magnesium. It contained no iron or sulfide and was rendered anoxic by bubbling with N_2 for 40 min.

Text S5. Rate conversion (% to mol).

H_2 concentration (%) was measured after each injection, time point for which there is a known total pressure ($P_{1.5\,bar}$). For each degree of saturation, and each H_2 injection interval, the rate r in percent per day was assessed based on linear fitting: $y = rx + b$, with (x, y) = (day, H_2 concentration). The rate r' in mmol.d^{-1}.g$^{-1}_{\text{sand-bentonite}}$ or in mmol.d^{-1}.cm$^{-3}_{\text{water}}$ was calculated as follow:

$$r' = \frac{(y_{inj}P_{1.5\,bar} - y_0 P_0) * V}{RT} * \frac{1}{x_0 - x_{inj}} * \frac{1}{V_{sand-bentonite}\ or\ V_{water}}$$

with R the ideal gas constant, T the temperature (20°C, 293 K), x_{inj} the day of H_2 injection, y_{inj} the H_2 concentration measured after the injection, (x_0, y_0) corresponding to the day where H_2 concentration reaches 0%: $x_0 = -\frac{b}{r}; y_0 = 0$, $V_{sand-bentonite} or\ V_{water}$ the bulk volume of sand-bentonite (for the solid-phase normalized calculation), or the volume of water in the vial (for the water normalized calculation), V the gas volume in the vial (known constant, Table S2), P_0, the total pressure when H_2 concentration reaches 0% (unknown but not required, as it cancels out as $y_0 = 0$).

2. Supplementary figures

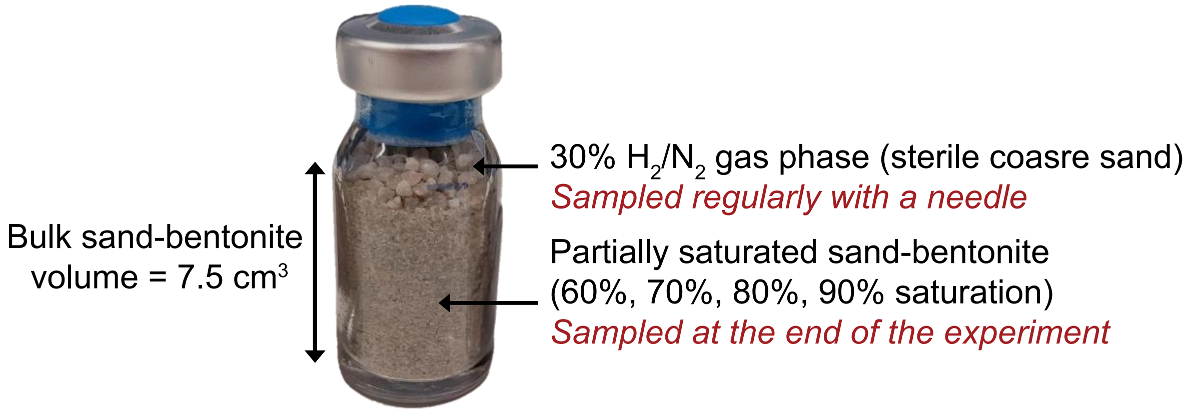

Figure S1. Experimental setup, vials filled with 7.5 cm^3 of partially saturated sand-bentonite with coarse sand layer on top to maintain a headspace.

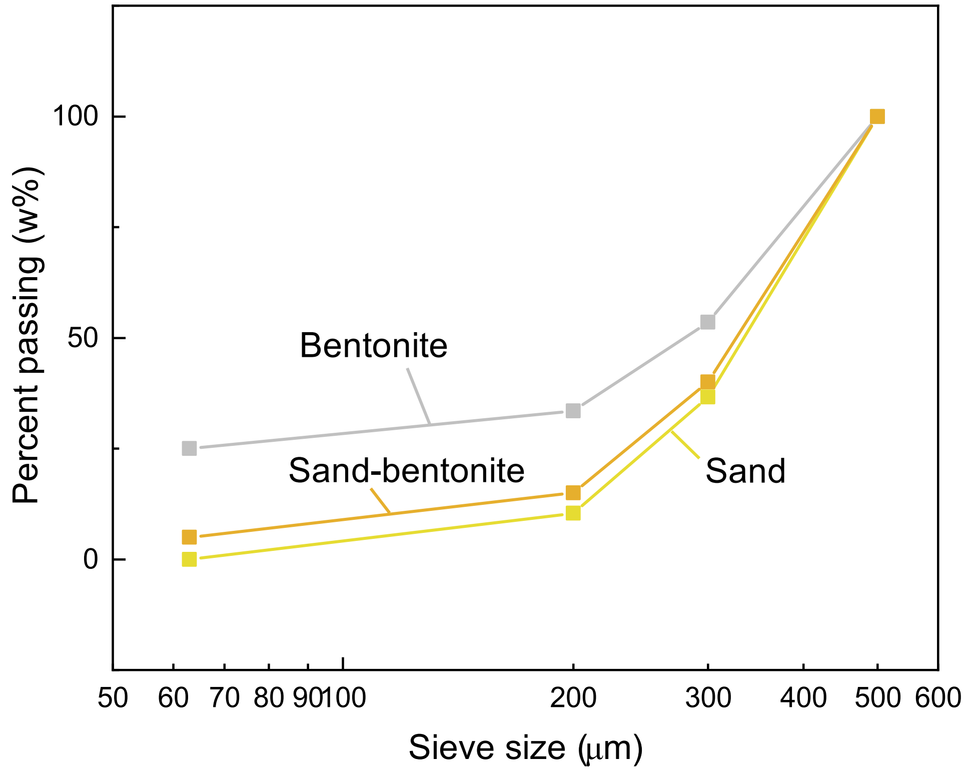

Figure S2. Sand-bentonite grain size distribution used in this experiment.

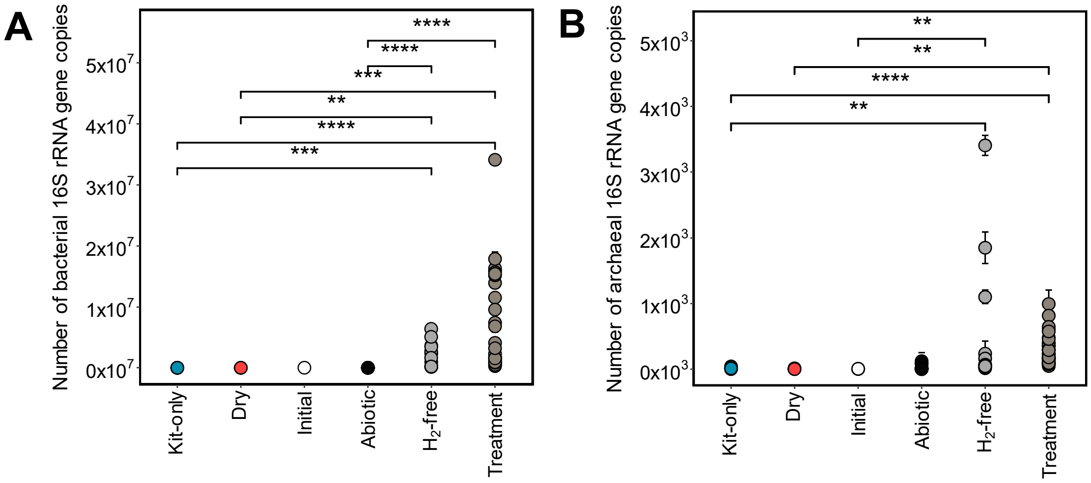

Figure S3. Number of (A) bacterial and (B) archaeal 16S rRNA gene copies per sand-bentonite sample (0.2 g) under different conditions (dry, initial, abiotic, H_2-free, and treatment with H_2), as well as in kit-only extractions. Stars show statistically significant difference between two conditions indicated with the bracket (Wilcoxon test, **: $p < 0.01$, ***: $p < 0.001$, ****: $p < 0.0001$).

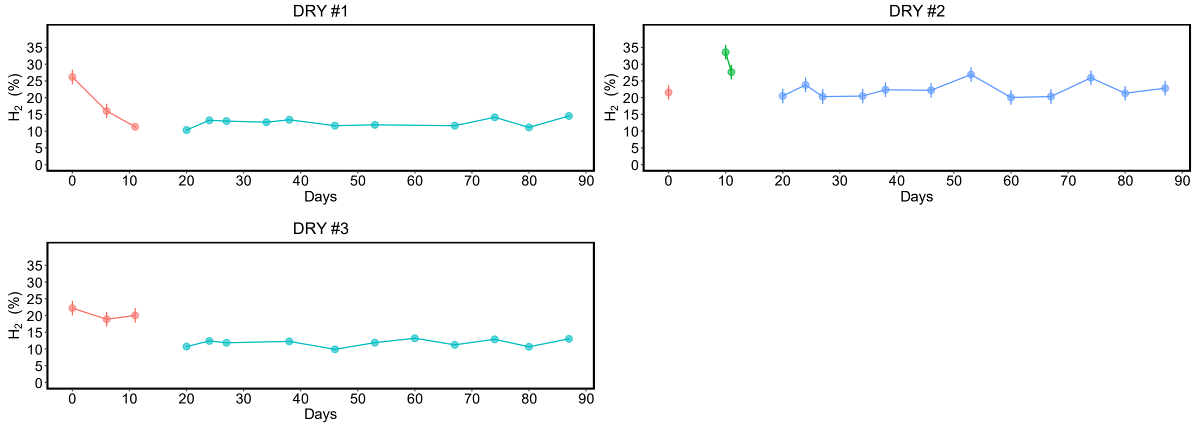

Figure S4. H_2 concentration in dry vials. Each injection is indicated by a different color and by connected data points.

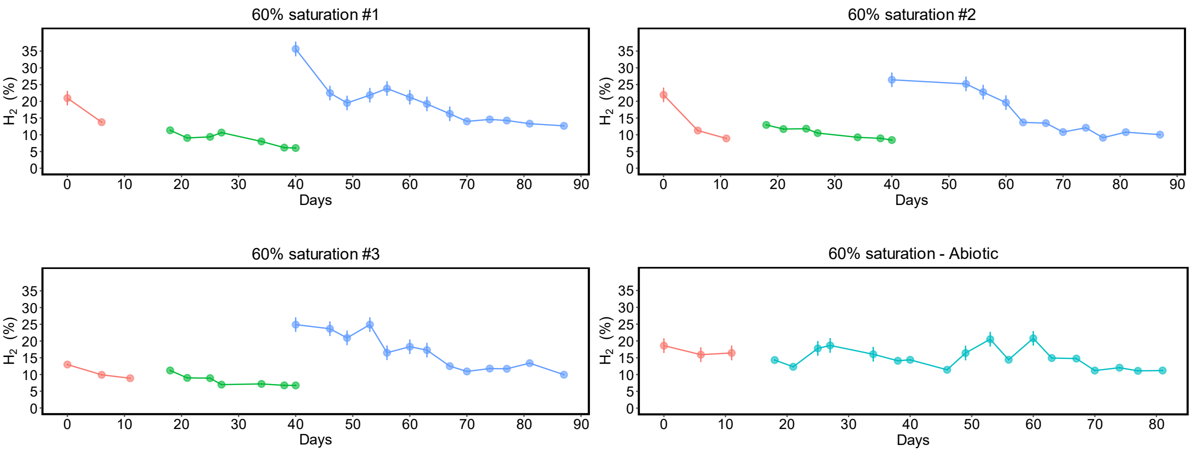

Figure S5. H_2 concentration in the 60% saturation treatment vials #1, 2, 3 and abiotic control. Each injection is indicated by a different color and by connected data points.

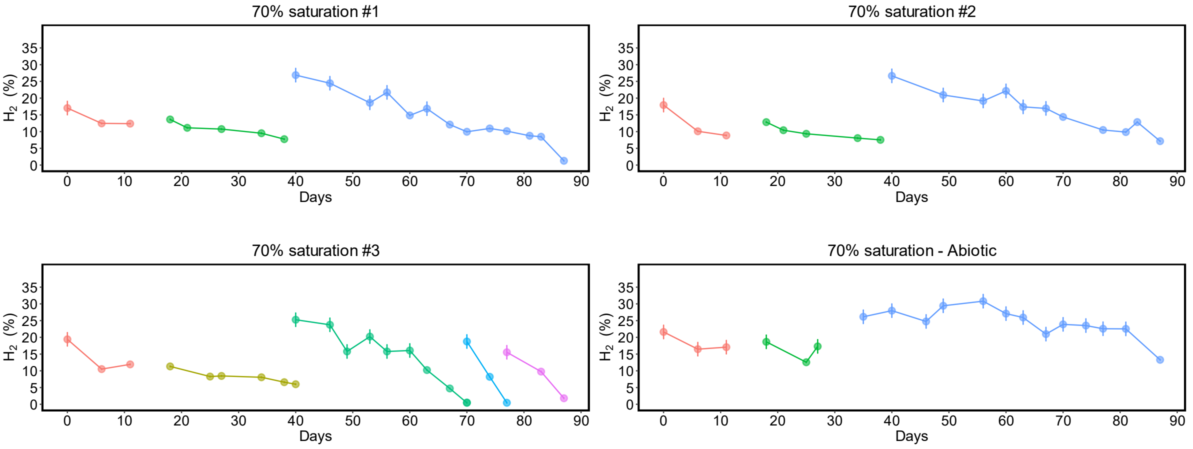

Figure S6. H_2 concentration in the 70% saturation treatment vials #1, 2, 3 and abiotic control. Each injection is indicated by a different color and by connected data points.

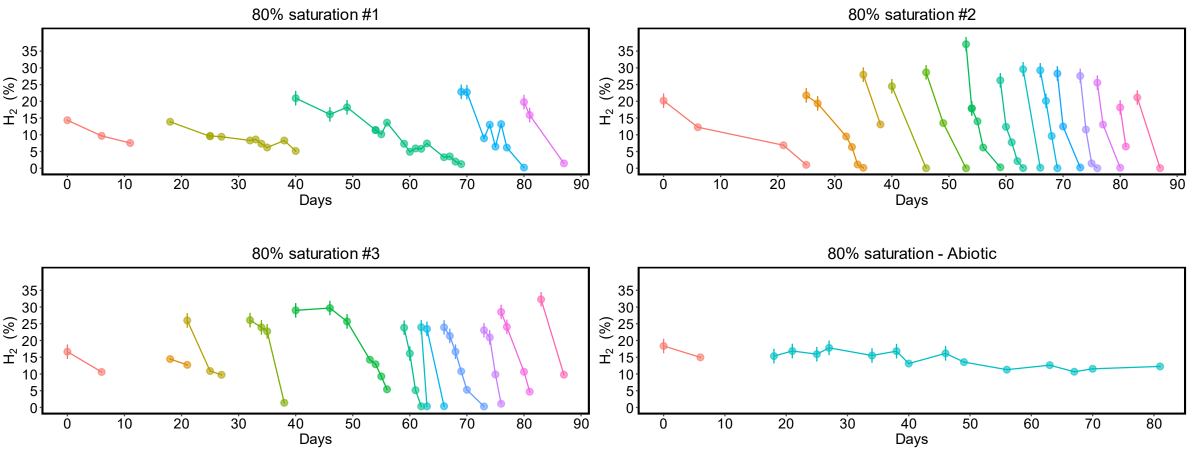

Figure S7. H_2 concentration in the 80% saturation treatment vials #1, 2, 3 and abiotic control. Each injection is indicated by a different color and by connected data points.

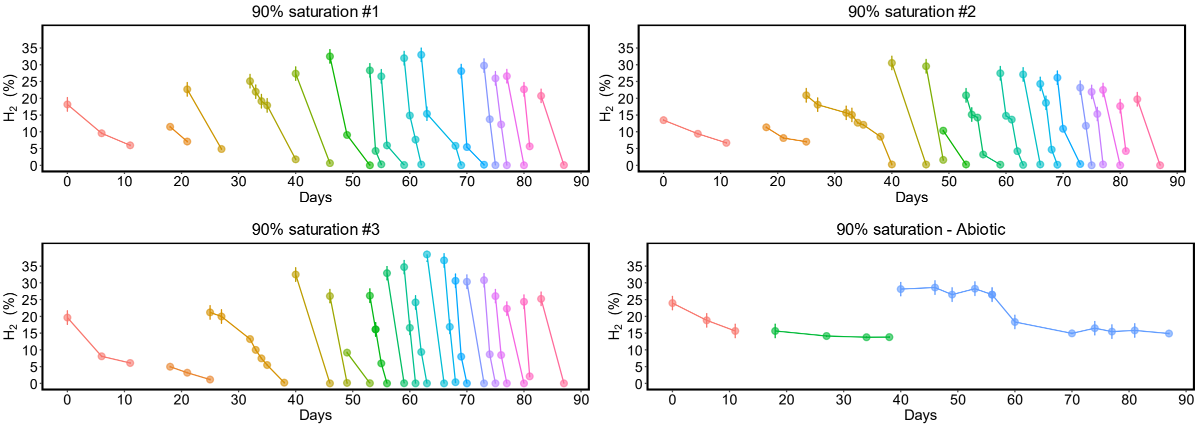

Figure S8. H_2 concentration in the 90% saturation treatment vials #1, 2, 3 and abiotic control. Each injection is indicated by a different color and by connected data points.

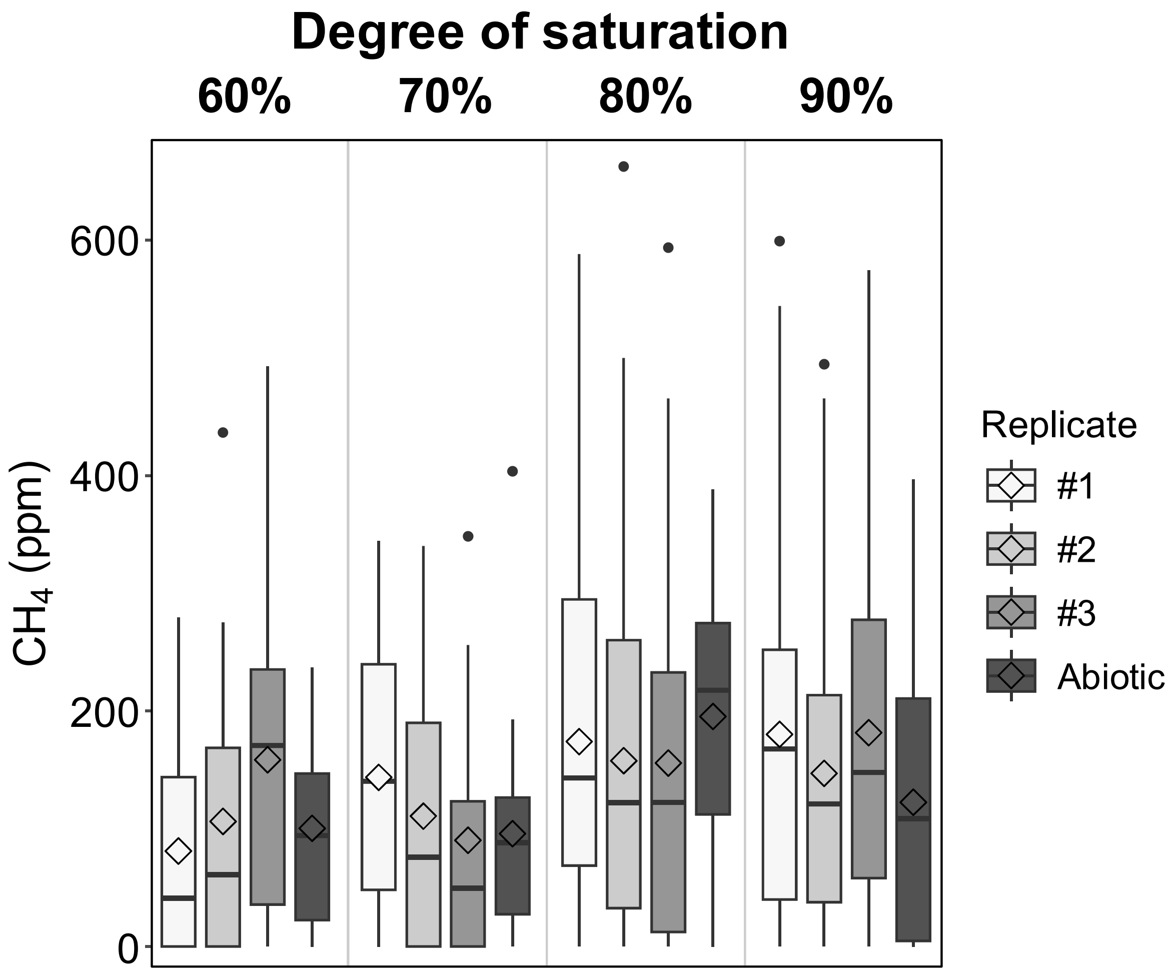

Figure S9. Box plot illustrating the CH_4 concentration over the course of the experiment, in treatment and abiotic vials for each saturation. The central line of each box represents the median, and the box edges correspond to the first and third quartiles. Whiskers extend to the minimum and maximum values within 1.5 times the interquartile range, while individual points beyond this range are plotted as outliers. The mean of each distribution is indicated by a diamond inside the box.

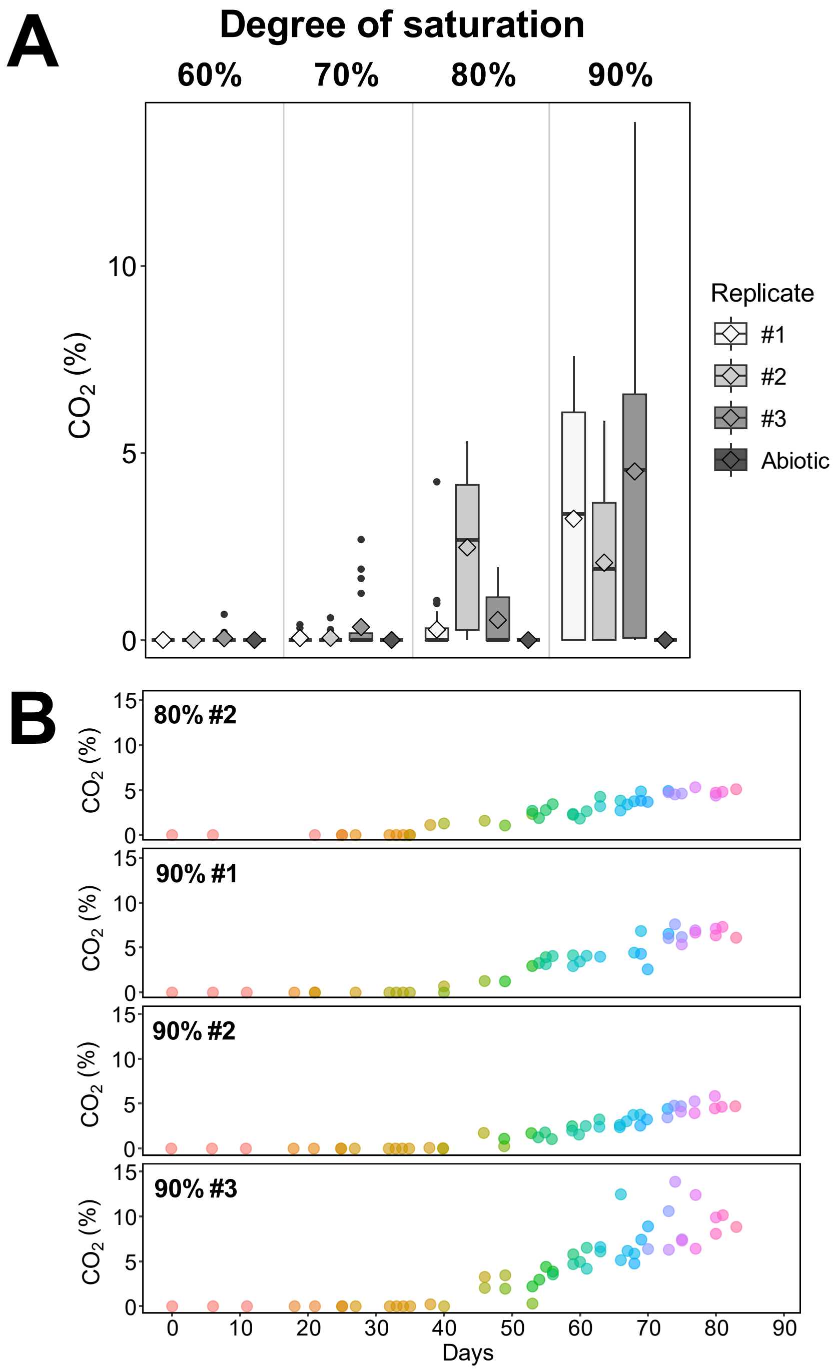

Figure S10. (**A**) Box plot illustrating CO_2 concentration over the course of the experiment, in treatment and abiotic vials for each saturation. The central line of each box represents the median, and the box edges correspond to the first and third quartiles. Whiskers extend to the minimum and maximum values within 1.5 times the interquartile range, while individual points beyond this range are plotted as outliers. The mean of each distribution is indicated by a diamond inside the box. No CO_2 was detected in the abiotic and treatment vials at 60% saturation, and 70% saturation vials #1 and #2. (**B**) Temporal evolution of CO_2 concentration in the vials with the highest detected levels. Color change corresponds to H_2 injections.

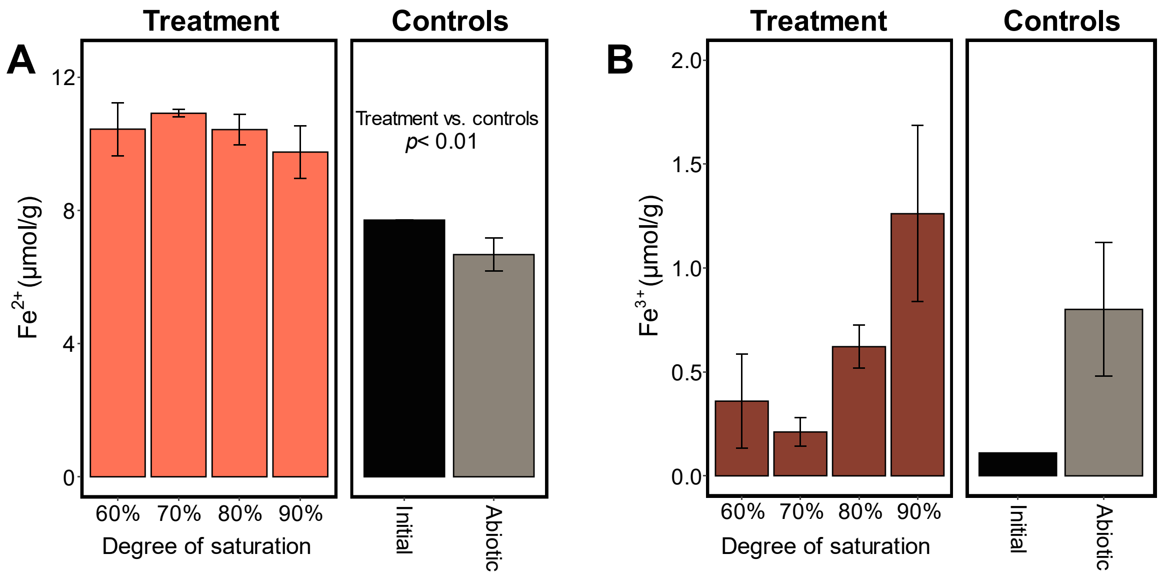

Figure S11. (A) Ferrous and (B) ferric iron extracted using 1M HCl (average of all vials for each degree of saturation). Error bars represent technical and experimental replicates. Kruskal-Wallis test results are annotated when significant, indicating that ferrous iron is significantly higher in treatment vials with H_2 compared to controls.

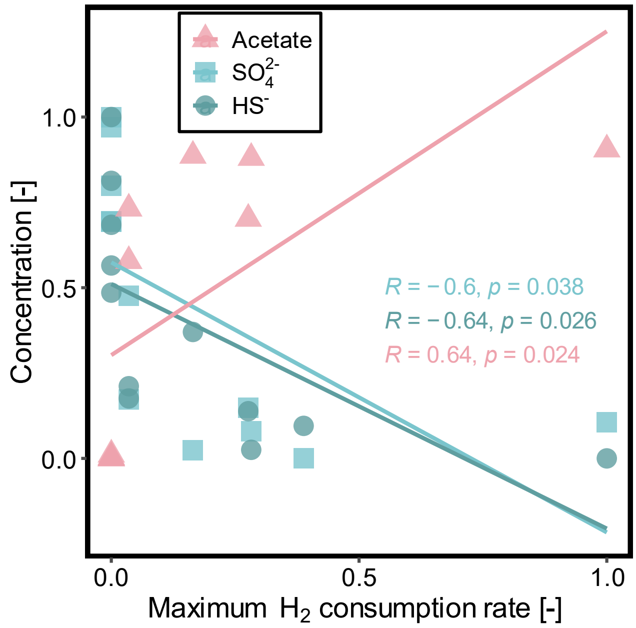

Figure S12. Correlation of acetate, sulfate, and sulfide ($\mu mol.g^{-1}$) measured at the end of the experiment with the maximum H_2 consumption rate ($\mu mol.d^{-1}.cm^{-3}_{sand\text{-}bentonite}$). Each point corresponds to a treatment vial. Data were normalized (min-max) prior to the regression and are presented unitless.

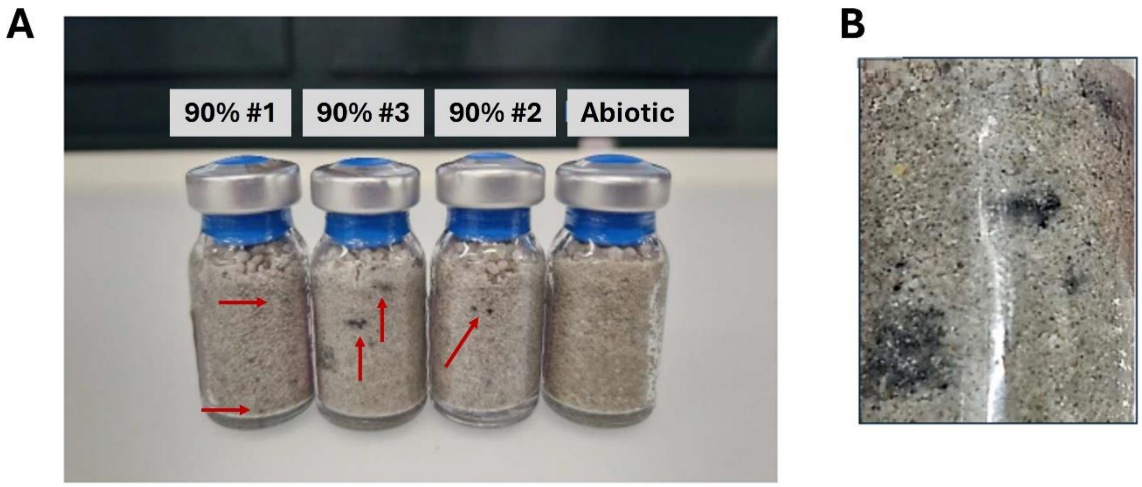

Figure S13. (A) Black spot formation (arrows) observed in all treatment vials at 90% saturation, along with the noticeable grayish coloration of the sand-bentonite mixture compared to the initial orange color in the abiotic control. (B) A close-up of vial #3 shows diffuse black spots with heterogeneous color.

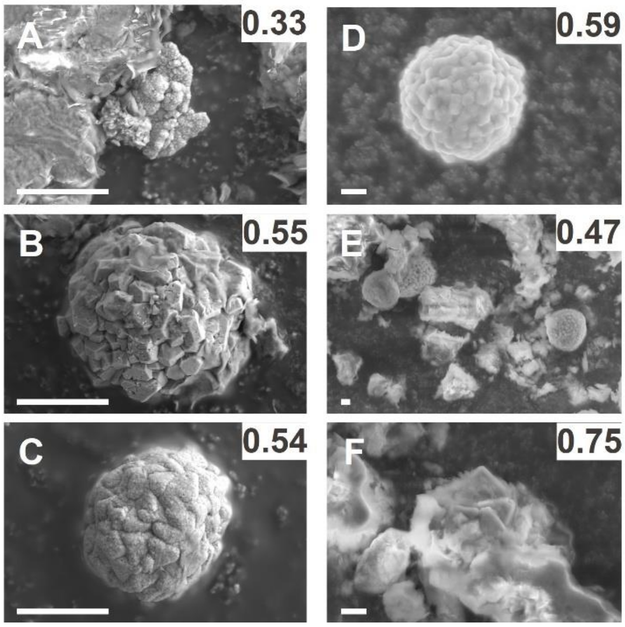

Figure S14. SEM images of iron-sulfide precipitates in vial #2 (**A–C**) and vial #3 (**D–F**) at 90% saturation, with the scale bar representing 1 µm. The iron:sulfur ratio, determined by EDS, is indicated within the white frame. All precipitates exhibit a framboidal shape, except for (**F**). Variations in framboid appearance are also observed. In vial #3, the framboids are notably larger and more developed than in vial #2.

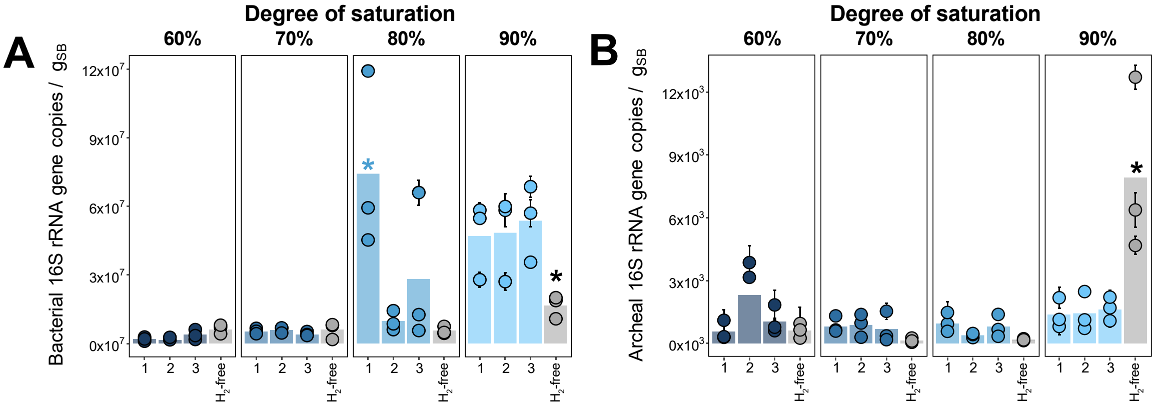

Figure S15. (A) Bacterial and (B) archaeal biomass quantification based on 16S rRNA gene qPCR (per gram of sand-bentonite (SB)), vials are grouped by degree of saturation. Points represent individual extractions, with error bars indicating technical replicates, the bar represents the average for each vial. For a certain degree of saturation: black stars indicate that the H_2-free control is significantly different than treatment vials, and blue stars indicate that a treatment vial is significantly different from the other treatment and abiotic vials.

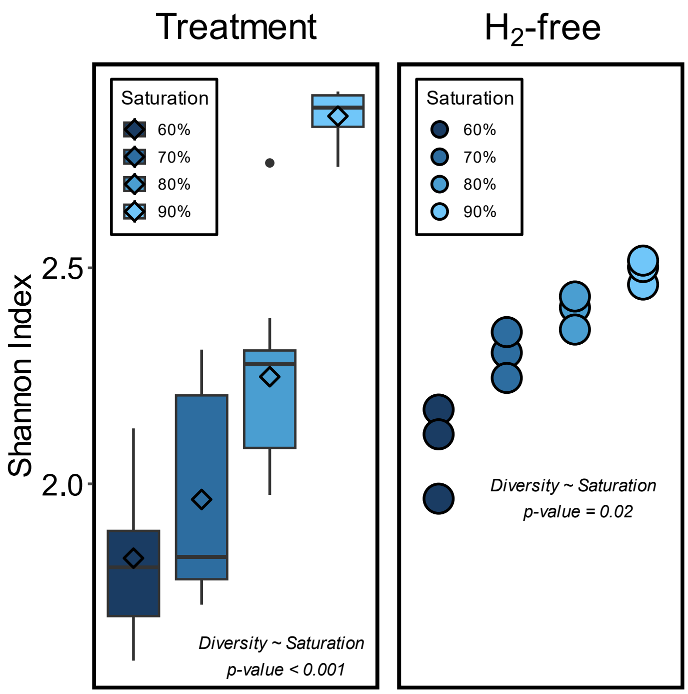

Figure S16. Alpha diversity (measured by the Shannon Index) across saturation levels in treatment vials with H_2 (left, boxplot) and in H_2-free controls (right, scatter plot). For the boxplot, the central line of each box represents the median, and the box edges correspond to the first and third quartiles. Whiskers extend to the minimum and maximum values within 1.5 times the interquartile range, while individual points beyond this range are plotted as outliers. The mean is indicated by a diamond inside the box. Kruskal-Wallis test results are annotated, showing that increasing water levels significantly enhance alpha diversity in both conditions. With H_2 treatment at 90% saturation, the 3 vials had a similar index, as indicated by the small box. Detailed pairwise tests are in Table S8.

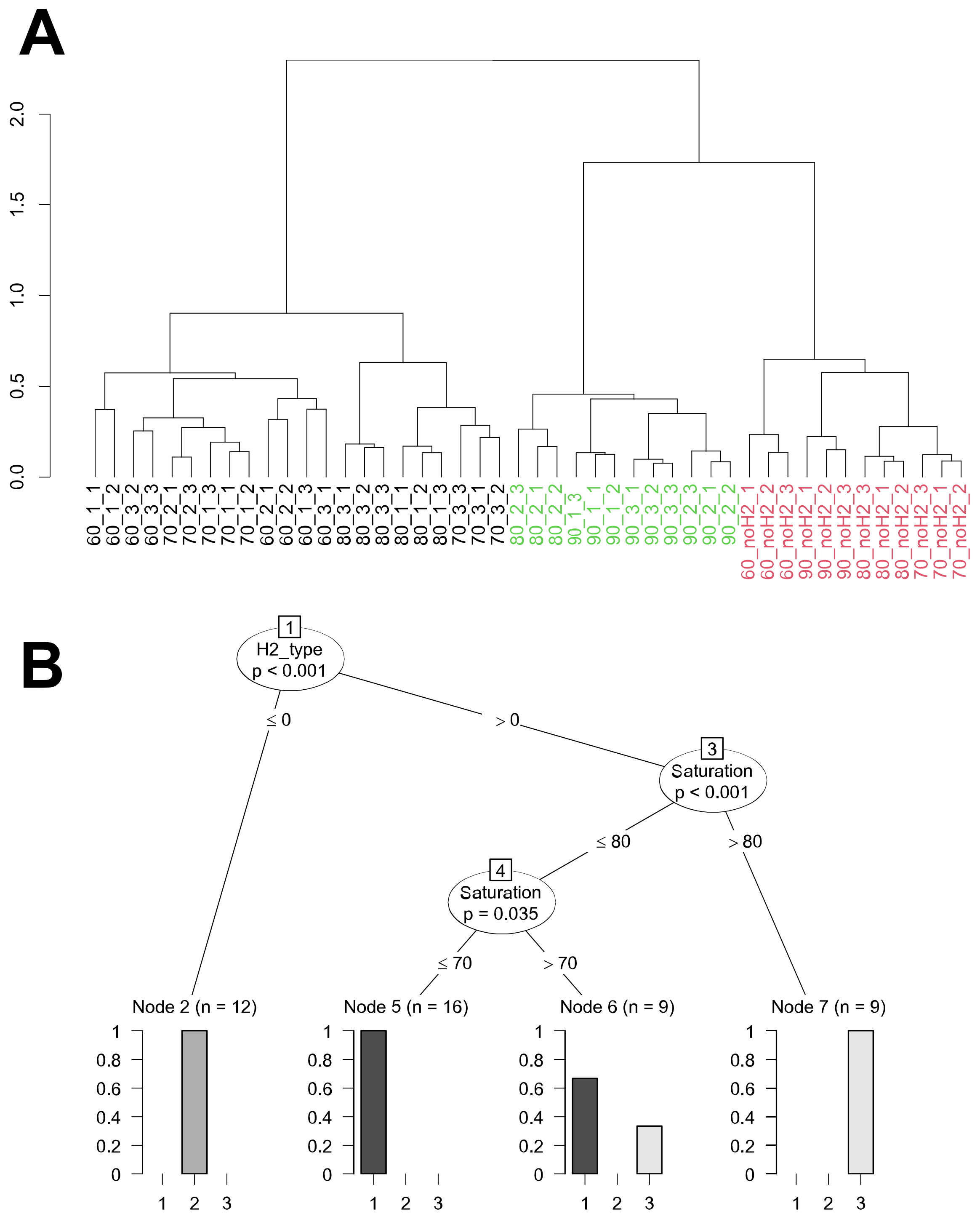

Figure S17. (A) Hierarchical clustering: tree built with Ward method; samples are colored according to clusters determined with the silhouette index. Each sample is labeled as "saturation_vial number_DNA extraction replicate" and colored according to its cluster. (B) Conditional inference tree predicting (A) clusters based on water and H_2 availability. Three threshold levels are identified: (1) H_2 presence/absence ($p < 0.001$), (2) 90% saturation ($p = 0.001$), and (3) 80% saturation ($p = 0.035$).

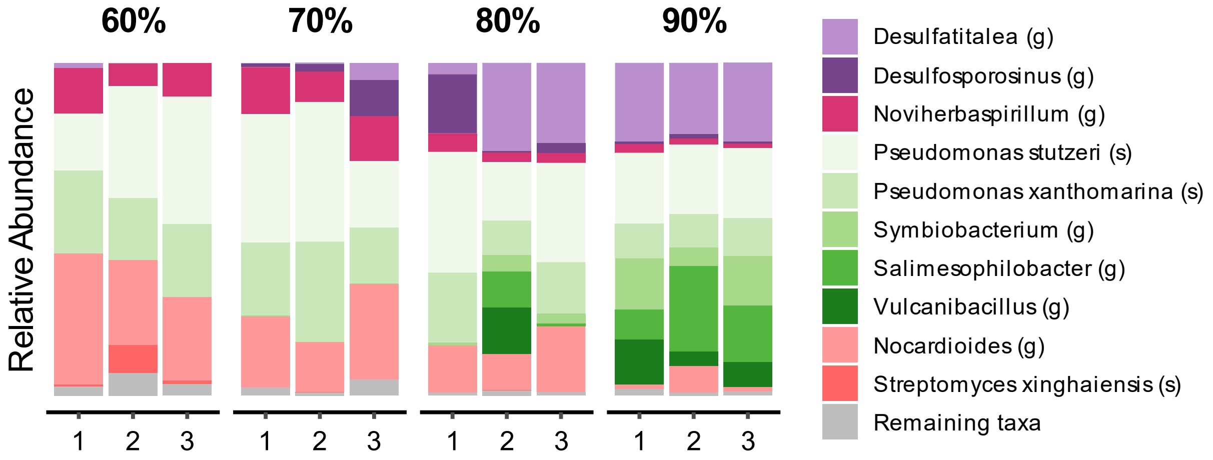

Figure S18. Relative abundance of taxa in H_2 treatment vials. Vials are organized by saturation level (60%, 70%, 80%, 90%). The relative abundance displayed is the average of triplicate extractions in each vial. ASVs are grouped by the lower taxonomic level assigned. Colors refer to taxa type: H_2 oxidizing sulfate reducers (purple), the H_2 oxidizing nitrate reducing and facultative anaerobic genus *Noviherbaspirillum* [2] (pink), anaerobic non H_2 oxidizing organoheterotrophs (green), aerobes (red).

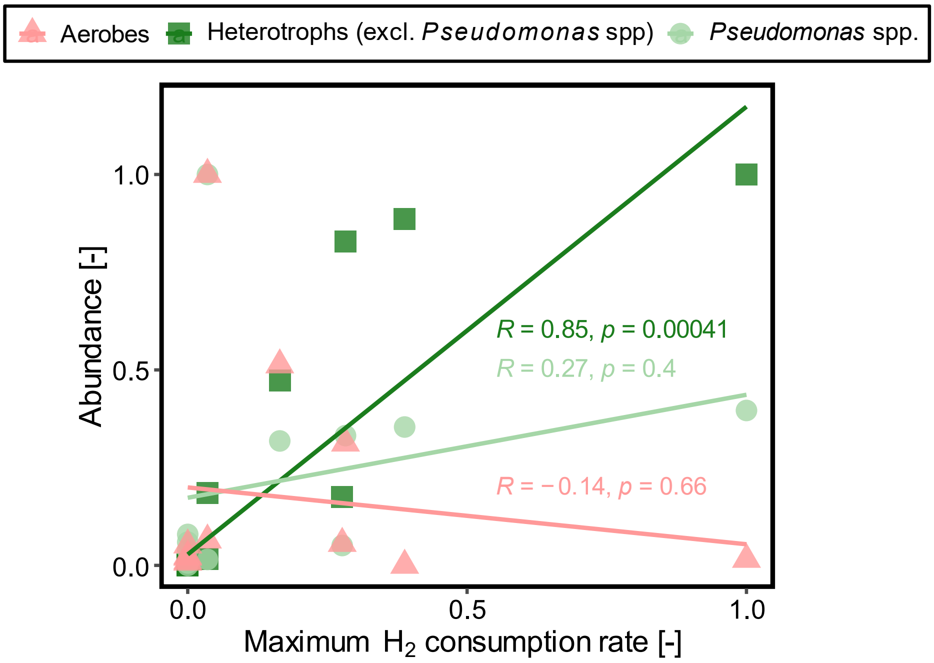

Figure S19. Correlation of H_2 consumption rate ($\mu mol.d^{-1}.cm^{-3}_{sand\text{-}bentonite}$) with aerobes, anaerobic non H_2 oxidizing organoheterotrophs excluding *Pseudomonas* spp., and *Pseudomonas* spp. abundance (16S rRNA copies.$g^{-1}_{sand\text{-}bentonite}$). Each point corresponds to a treatment vial. Data were normalized (min-max) prior to the regression and are presented unitless.

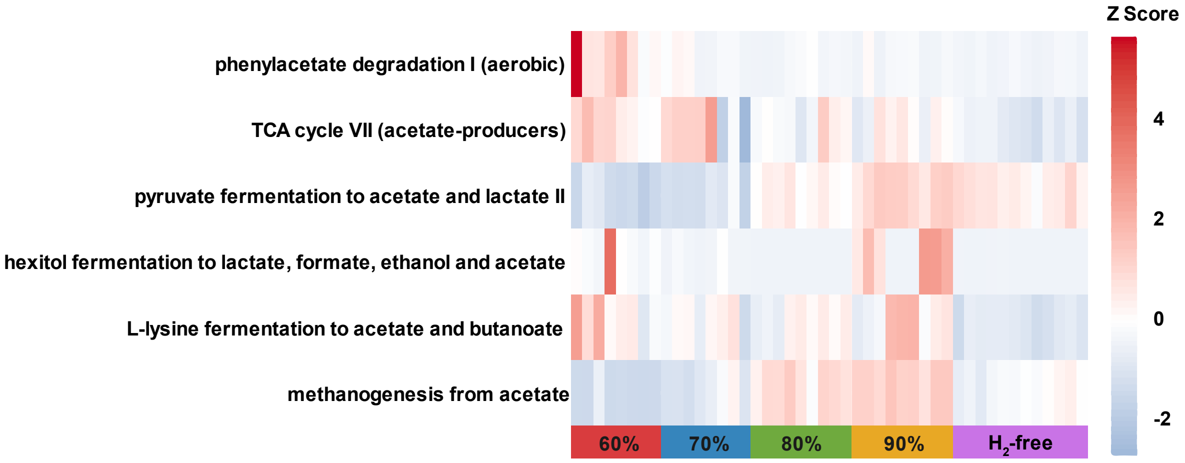

Figure S20. Heatmap of z-scores for acetate-generating and -consuming pathways predicted using PICRUSt for each sample. Samples are grouped by saturation level (60%, 70%, 80%, 90%) for the H_2 treatment, while all samples from the H_2-free controls are grouped together.

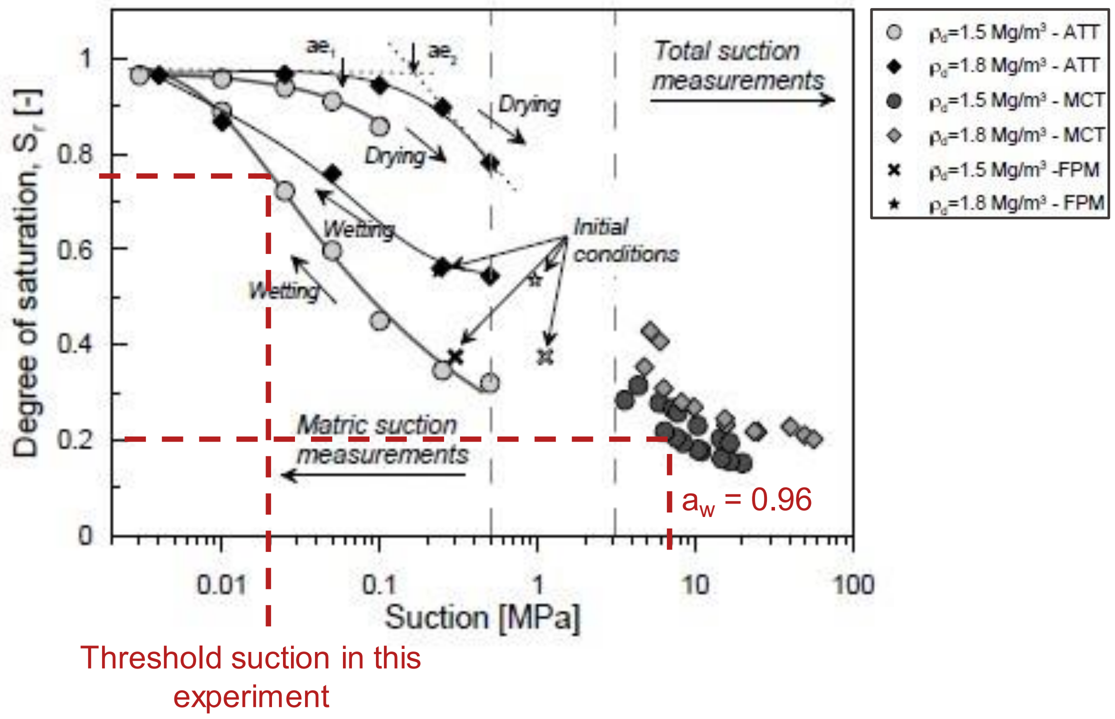

Figure S21. Sand-bentonite water retention curve for two dry densities (1.5 g.cm^{-3} and 1.8 g.cm^{-3}), adapted with permission from Manca [1]. Copyright 2016 Nagra. The saturation level expected to be a threshold for microbial growth (water activity of 0.96) and the observed threshold in this study (70-80%) are highlighted. Total or matric suction was measured across the entire range using three techniques, selected based on best suitability for the conditions: Axis Translation Technique (ATT), Micro Cell Technique (MCT), and Filter Paper Method (FPM).

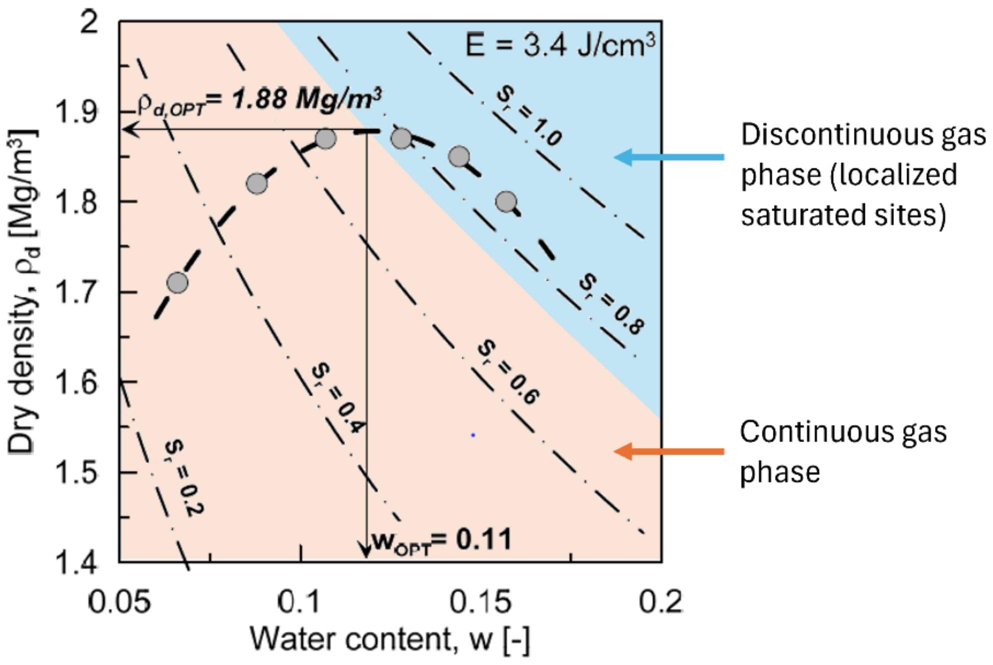

Figure S22. Dynamic compaction test (3.4 J.cm^{-3}) on sand-bentonite, adapted with permission from Manca [1]. Copyright 2016 Nagra. Each compaction test (water content and dry density achieved) is indicated by a grey point. Maximum density is achieved at the transition saturation between a continuous gas phase and a non-continuous gas phase with water pockets.

3. Supplementary tables

Table S1. Detailed values for the sand-bentonite mass and volumes, and water volumes used for the preparation of the vials.

DSr (%)	Sand-bentonite mass (g)	Initial water content (-)	Sand-bentonite dry dry (g)	Sand-bentonite bulk volume (cm^3)	Dry bulk density (g/cm^3)	Sand-bentonite solid volume (cm^3)	Voids volume (cm^3)	Water volume added (cm^3)	Total water volume (cm^3)
**60**	11.5	3	11.2	7.5	1.5	4.2	3.3	1.7	2.0
**70**	11.5	3	11.2	7.5	1.5	4.2	3.3	2.0	2.3
**80**	11.5	3	11.2	7.5	1.5	4.2	3.3	2.3	2.7
**90**	11.5	3	11.2	7.5	1.5	4.2	3.3	2.7	3.0

Table S2. The water and gas volumes (after water injection) for each degree of saturation. The water volume includes both the added water, and the water initially present in the material (Table S1), determined by oven drying at 105°C for 24 h. The gas volume was calculated from the volume required to generate a 0.5 bar overpressure in the vial and thus include the gas in the sand-bentonite porosity and in the headspace.

DSr (%)	Water (cm^3)	Gas (cm^3)
**60**	2.0	0.93
**70**	2.3	0.84
**80**	2.7	0.75
**90**	3.0	0.67

Table S3. Suction measurements of sand-bentonite samples wetted with artificial formation water after one week of equilibration. The reported water activity (a_w) corresponds to the total suction measured (Ψ): $\Psi = -\frac{RT\rho}{\omega}\ln(a_w)$ where R is the universal gas constant, T the temperature, ρ the density of water, and ω the molecular mass of water.

DSr (%)	Total suction (Mpa)	Water activity (-)
67	2.5	0.982
74	2.0	0.986
78	1.7	0.988
81	1.6	0.988
84	1.5	0.989

Table S4. New protocol for DNA extraction from clayey materials. The Qiagen PowerSoil Pro Kit protocol was iteratively optimized by testing the following modifications: (1) adjusting the hexametaphosphate (HMP) concentration (5% or 10%), (2) adding HMP either with the lysis solution or prior to lysis, for the later followed by removal by centrifugation, (3) varying the volume of HMP used (100, 200, or 300 µL), and (4) incubating the DNA eluent on the filter for different durations (0, 5, 10, or 15 min). Each iteration was tested on 0.2 g sand-bentonite (SB) samples and kit-only (kit reagents alone), with selected parameters carried forward to subsequent tests. DNA yield was measured with Qubit 1x dsDNA HS Assay Kit.

Test	Sample	Treatment	DNA (ng/mL)	Notes
(1) HMP concentration	SB	no HMP (unmodified protocol)	5.00	
	SB	+5%HMP	497.00	
	**SB**	**+10%HMP**	**672.00**	selected for tests (2-4)
	Kit-only	no HMP	< loq	
	Kit-only	+5%HMP	10.30	
	Kit-only	+10%HMP	8.30	
(2) Addition of HMP with or before lysis buffer	SB	before lysis	157 ± 5	
	**SB**	**with lysis**	**180 ± 25**	selected for tests (3-4)
(3) Volume of HMP	SB	100 uL	456.00	
	**SB**	**200 uL**	**574.00**	selected for test (4)
	SB	300 uL	420.00	
(4) DNA eluent incubation (room temperature)	SB	0 min	398.00	
	SB	5 min	652.00	
	**SB**	**10 min**	**884.00**	selected
	SB	15 min	772.00	
	Kit-only	10 min	50 ± 10	

Table S5. Average H_2 consumption rate in treatment vials excluding the first 35 days, normalized by bulk sand-bentonite or water volume. Standard deviation is annotated except for 60% saturated vials, and 70% #1 and #2, for which a single rate was measured. The average for each degree of saturation is also reported.

**Sat.**	**Vial**	**Rate ($\mu mol.d^{-1}.cm^{-3}_{water}$)**		**Rate ($\mu mol.d^{-1}.cm^{-3}_{sand\text{-}bentonite}$)**	
**60%**	**1**	0.09	0.08 ± 0.01	0.02	0.02 ± 0.00
	**2**	0.08		0.02	
	**3**	0.07		0.02	
**70%**	**1**	0.07	0.13 ± 0.09	0.02	0.04 ± 0.03
	**2**	0.06		0.02	
	**3**	0.26 ± 0.17		0.08 ± 0.05	
**80%**	**1**	0.23 ± 0.13	0.83 ± 0.47	0.08 ± 0.05	0.29 ± 0.17
	**2**	1.39 ± 0.85		0.49 ± 0.30	
	**3**	0.86 ± 0.46		0.30 ± 0.16	
**90%**	**1**	1.71 ± 1.52	1.60 ± 0.24	0.68 ± 0.61	0.63 ± 0.10
	**2**	1.26 ± 1.10		0.50 ± 0.44	
	**3**	1.82 ± 1.04		0.72 ± 0.42	

Table S6. Pairwise t-tests performed on sulfate, sulfide, and acetate concentrations at the end of the experiment, with p-values adjusted using the Benjamini-Hochberg method. Treatment vials were grouped by degree of saturation. Significant p-value are highlighted.

Sulfate t-test					
	**60%**	**70%**	**80%**	**90%**	**Abiotic**
**70%**	0.942	-	-	-	-
**80%**	**0.031**	**0.032**	-	-	-
**90%**	**0.009**	**0.009**	0.463	-	-
**Abiotic**	**0.009**	**0.009**	**0.000**	**0.000**	-
Sulfide t-test					
	**60%**	**70%**	**80%**	**90%**	**Abiotic**
**70%**	0.537	-	-	-	-
**80%**	0.145	0.469	-	-	-
**90%**	**0.005**	**0.015**	0.053	-	-
**Abiotic**	0.191	0.583	0.759	**0.030**	-
Acetate t-test					
	**60%**	**70%**	**80%**	**90%**	**Abiotic**
**70%**	0.155	-	-	-	-
**80%**	**0.000**	**0.000**	-	-	-
**90%**	**0.000**	**0.000**	0.188	-	-
**Abiotic**	0.796	0.188	**0.000**	**0.000**	-

Table S7. Pairwise Wilcoxon test performed on results of Bacterial and Archaeal qPCR 16S rRNA gene. Treatment vials were grouped by degree of saturation, while control vials were categorized as either abiotic or H_2-free. NS: not significant.

**Bacteria qPCR - Wilcoxon test**						
	**60%**	**70%**	**80%**	**90%**	**H_2-free**	**Abiotic**
**60%**	-	**	***	****	NS	****
**70%**		-	***	****	NS	****
**80%**			-	NS	NS	****
**90%**				-	****	****
**H_2-free**					-	****
**Abiotic**						-
**Archaea qPCR - Wilcoxon test**						
	**60%**	**70%**	**80%**	**90%**	**H_2-free**	**Abiotic**
**60%**	-	NS	NS	NS	NS	***
**70%**		-	NS	NS	NS	***
**80%**			-	NS	NS	***
**90%**				-	NS	***
**H_2-free**					-	**
**Abiotic**						-

Table S8. Pairwise Wilcoxon tests performed on results of alpha diversity (Shannon index). Vials were grouped based on presence/absence of H_2, and degree of saturation. Significant p-value are highlighted.

		60%		70%		80%		90%
		H_2	H_2-free	H_2	H_2-free	H_2	H_2-free	H_2
60%	H_2-free	0.117	-	-	-	-	-	-
70%	H_2	0.353	0.630	-	-	-	-	-
	H_2-free	**0.026**	0.117	0.117	-	-	-	-
80%	H_2	**0.011**	0.316	0.117	0.622	-	-	-
	H_2-free	**0.026**	0.117	**0.026**	0.117	0.117	-	-
90%	H_2	**0.001**	**0.026**	**0.001**	**0.026**	**0.001**	**0.026**	-
	H_2-free	**0.026**	0.117	**0.026**	0.117	0.117	0.117	**0.026**

References

[1] D. Manca, “Technical Report 16-07 - Gas transport and related Chemo-Hydro-Mechanical response of sand bentonite mixture.” NAGRA, May 2016.

[2] S. Ishii *et al.*, “Noviherbaspirillum denitrificans sp. nov., a denitrifying bacterium isolated from rice paddy soil and Noviherbaspirillum autotrophicum sp. nov., a denitrifying, facultatively autotrophic bacterium isolated from rice paddy soil and proposal to reclassify Herbaspirillum massiliense as Noviherbaspirillum massiliense comb. nov.,” *Int. J. Syst. Evol. Microbiol.*, vol. 67, no. 6, pp. 1841–1848, June 2017, doi: 10.1099/ijsem.0.001875.
